# Supplementary material for: The relative impact of interventions on sympatric Plasmodium vivax and Plasmodium falciparum malaria: A systematic review
Source: PLoS Negl Trop Dis. 2022 Jun 29;16(6):e0010541. doi: 10.1371/journal.pntd.0010541 (PMC9242512; doi:10.1371/journal.pntd.0010541)
Supplement: S1 Supporting Information — (DOCX) [file pntd.0010541.s001.docx]

**S1 Supporting information:**

**Included studies, and characteristics of the series of time-points by intervention**

**Included studies**

Table A. Description of included studies

| **First author** | **Country** | **Study type** | **Number of series of time points**  **with patent infections** | **Number of series of time points with clinical cases** |
| --- | --- | --- | --- | --- |
| Ome-Kaius [1], Lin [2], Betuela [3] | Papua New Guinea | observational | 2 | 2 |
| Kattenberg [4] | Papua New Guinea | observational | 11 | - |
| Koepfli [5] | Papua New Guinea | observational | 2 | - |
| Graves [6] | Papua New Guinea | observational | - | - |
| Chaumeau [7], Landier [8] | Myanmar | observational | 4 | 3 |
| Rodriguez-Rodriguez [9] | Papua New Guinea | observational | - | 7 |
| Deressa [10] | Ethiopia | observational | 1 | - |
| Hetzel [11], Hetzel [12], Hetzel[13] | Papua New Guinea | observational | 10 | - |
| Hetzel [14] | Papua New Guinea | observational | 5 | 1 |
| Garfield [15] | Nicaragua | observational | - | 1 |
| Luxemburger [16] | Thai-Myanmar border | observational | 1 | - |
| Metselaar [17] | Indonesia | observational | 1 | - |
| Kessler [18] | India | observational | - | 5 |
| Loha [19] | Ethiopia | observational | - | 2 |
| Hanafi-Bojd [20] | Iran | observational | - | - |
| Mitja [21] | Papua New Guinea | observational | - | 1 |
| Sharma [22] | India | observational | 1 | - |
| Chaves [23], Kaneko [24] | Vanuatu | observational | - | 2 |
| Gabaldon [25] | Venezuela | observational | - | 2 |
| Kattenberg [26] | Vietnam | observational | - | 1 |
| Maude [27] | Cambodia | observational | - | 9 |
| Charlwood [28] | Brazil | observational | - | 2 |
| McGreevy [29] | Brazil | observational | 1 | 1 |
| Sena [30] | Ethiopia | observational | - | 4 |
| Hofmann [31], Robinson [32] | Papua New Guinea | observational | 2 | - |
| Thomson [33] | Cambodia | observational | 1 | - |
| Loha [34] | Ethiopia | trial | - | 2 |
| Doke [35] | India | observational | - | 1 |
| Kligler [36] | Palestine | observational | 3 | - |
| Van Dijk [37] | Indonesia | observational | 1 | - |
| Hii [38] | Solomon Islands | trial | 2 | - |
| Rowland [39] | Afghanistan | observational | 2 | - |
| Kondrashin [40] | India | observational | - | 12 |
| Sahu [41] | India | trial | - | 1 |
| Shah [42] | India | observational | - | 3 |
| Mishra [43] | India | observational | - | 1 |
| Rowland [44] | Pakistan | observational | 5 | - |
| Rowland [45] | Pakistan | observational | 8 | 2 |
| Rowland [46] | Pakistan | trial | 2 | 2 |
| Sluydts[47], Sluydts [48] | Cambodia | observational | 1 | - |
| Smithuis [49] | Myanmar | trial | 1 | - |
| Landier [50] | Myanmar | observational | - | 1 |
| Rowland [51], Rowland [52] | Pakistan | trial | 1 | 1 |
| Singh [53] | India | observational | - | 2 |
| Seyoum [54] | Ethiopia | observational | - | 1 |
| Lwin [55] | Thai-Myanmar border | observational | 1 | - |

## **Characteristics of the series of time-points**

### **Characteristics of the series of time-points: clinical cases**

Table B Characteristics of series of time-points: first-time ITN clinical cases

| **First author** | **Study area** | **Year of baseline** | **Number of data points** | **Relapse pattern** | **Sea-son-ality** | **Trans-**  **mission**  ***Pf Pv*** | **Coverage** | **Diag-**  **nostic**  **tool** | **Mixed cases** | **Age groups** | **Total number of cases found in series of time-points** |
| --- | --- | --- | --- | --- | --- | --- | --- | --- | --- | --- | --- |
| Ome-Kaius [1], Lin [2], Betuela [3] | Papua New Guinea, Ilahita area | 2006 | 2 | frequent | low | high high | high | LM | not reported | Study population below 55 months | 2114 |
| Hetzel [14] | Papua New Guinea, Dreikikir,Mumeng, Sausi | 2008 | 2 | frequent | low | low high | low | LM | not reported | Median age of people in study: 2005: 17, 2013: 22 | 897 |
| Kessler^1^ [18] | India, Meghalaya, west khasi hills | 2016 | 5 | long | high | low low | low | missing | not reported | Around 15-19% of cases in under 5 -year-olds | 4613 |
| Kessler^1^ [18] | India, Meghalaya, south garo hills | 2016 | 5 | long | high | low low | low | missing | not reported | Around 20-25% of cases in under 5-year-olds | 22791 |
| Kessler^1^ [18] | India, Meghalaya, ri bhoi | 2016 | 5 | long | high | low low | low | missing | not reported | 11-17% of cases in under 5-year-olds | 989 |
| Kessler^1^ [18] | India, Meghalaya, jaintia hills | 2016 | 5 | long | high | low low | low | missing | not reported | 14-21% of cases in under 5-year-olds | 2765 |
| Kessler^1^ [18] | India, Meghalaya, east garo hills | 2016 | 5 | long | high | low low | low | missing | not reported | 22-26% of cases in under 5-year-olds | 14269 |
| Chaves^2^ [23], Kaneko [24] | Vanuatu | 1992 | 4 | frequent | low | low low | low | LM | not reported | Peak of malaria in 3-4 year olds (*Pv* and *Pf*) | 43502 |
| Sahu [41] | India, Orissa state, Malkangiri district | 1998 | 4 | both | high | high low | low | LM | given separately but could be with *Pm* but because few cases of *Pm* assumed to be mixed *Pv* and *Pf* | Peak incidence of malaria in 1-4 year olds | 255 |
| Shah^3^ [42] | India, Sundargarh district, village: JD | 2008 | 2 | both | high | high low | high | LM | most likely counted towards *falciparum* | 32% of cases in under 4-year-olds before intervention, 18% after intervention | 232 |
| Shah^3^ [42] | India, Sundargarh district, village: PP | 2008 | 2 | both | high | high low | high | LM | most likely counted towards *falciparum* | 32% of cases in under 4-year-olds before intervention, 18% after intervention | 306 |
| Shah^3^ [42] | India, Sundargarh district, village: TM | 2008 | 2 | both | high | high low | high | LM | most likely counted towards *falciparum* | 32% of cases in under 4-year-olds before intervention, 18% after intervention | 530 |
| Rowland [51], Rowland [52] | Pakistan, Mardan district, Baghicha and Kagan | 1991 | 4 | long | low | low high | high | LM | not reported | Mean age of population 19 | 1172 |
| Chaumeau [7], Landier [8] | Myanmar, Kayin State, B1-TPN | 2013 | 9 | frequent | high | low high | high | RDT or LM | counted towards both | Median age of population 20 | 64 |
| Loha [34] | Ethiopia, Adami Tullu | 2015 | 2 | long | low | low low | low | RDT | given separately | 19% of population under 5-year-olds | 678 |
| Maude^4^ [27] | Cambodia, Battambang | 2009 | 5 | frequent | high | low low | high | RDT or LM | not reported | Not reported | 1180 |
| Maude^4^ [27] | Cambodia, Kampot and Kampong Speu province | 2009 | 5 | frequent | high | low low | high | RDT or LM | not reported | Not reported | 2221 |
| Maude^4^ [27] | Cambodia, Kratie province | 2009 | 5 | frequent | high | low low | high | RDT or LM | not reported | Not reported | 1506 |
| Maude^4^ [27] | Cambodia, Pailin Province | 2009 | 5 | frequent | high | high high | high | RDT or LM | not reported | Not reported | 532 |
| Maude^4^ [27] | Cambodia, Preah Vihear Province | 2009 | 5 | frequent | high | high low | high | RDT or LM | not reported | Not reported | 1948 |
| Maude^4^ [27] | Cambodia, Pursat Province | 2009 | 5 | frequent | high | low low | high | RDT or LM | not reported | Not reported | 1195 |
| Maude^4^ [27] | Cambodia, Rattanakiri Province | 2009 | 5 | frequent | high | low low | high | RDT or LM | not reported | Not reported | 2362 |
| Maude^4^ [27] | Cambodia, Sen Monorom Province | 2009 | 5 | frequent | high | high low | high | RDT or LM | not reported | Not reported | 816 |
| Maude^4^ [27] | Cambodia, Steung Treng Province | 2009 | 5 | frequent | low | low low | high | RDT or LM | not reported | Not reported | 966 |
| Sena [30] | Ethiopia, gilgel gibe hydroelectric dam | 2005 | 5 | long | low | low low | missing | missing | not reported | 10.5% of cases in under 5-year-olds | 19789 |
| Sena [30] | Ethiopia, gilgel gibe hydroelectric dam, control area | 2005 | 5 | long | low | low low | missing | missing | not reported | 12.7% of cases in under 5-year-olds | 49879 |

*^1^case numbers were aggregated by season to decrease stochasticity. ^2^net distribution determined from figure 3A in Chaves[23], using the highest peaks (1992), relapse pattern assumed to be the same as in PNG, data added as yearly average  ^3^only percentages of cases caused by P. falciparum given. Remaining were assumed to be caused by P. vivax although there were some P. malariae but only very few ^4^figure 9b in Maude [27] was used to determine when nets were implemented*.

Table C Characteristics of series of time-points: repeated time ITN clinical cases

| **First author** | **Study area** | **Year of baseline** | **Number of data points** | **Relapse pattern** | **Sea-son-**  **ality** | **Trans-mission *Pf* *Pv*** | **Coverage** | **Diag-nostic tool** | **Mixed cases** | **Age groups** | **Total number of cases found in series of time-point** |
| --- | --- | --- | --- | --- | --- | --- | --- | --- | --- | --- | --- |
| Rodriguez-Rodriguez [9] | Papua New Guinea, Arawa | 2013 | 2 | frequent | low | low low | low | RDT then LM | given separately | Peak of malaria in 0-4 year olds | 254 |
| Rodriguez-Rodriguez [9] | Papua New Guinea, Dreikikir | 2012 | 3 | frequent | low | low low | high | RDT then LM | given separately | Peak of malaria in 0-4 year olds | 761 |
| Rodriguez-Rodriguez [9] | Papua New Guinea, East Cape | 2010 | 4 | frequent | low | high low | high | RDT then LM | given separately | Peak of malaria in 0-4 year olds | 3347 |
| Rodriguez-Rodriguez [9] | Papua New Guinea, Lemakot | 2012 | 3 | frequent | low | high low | low | RDT then LM | given separately | Peak of malaria in 0-4 year olds | 2937 |
| Rodriguez-Rodriguez [9] | Papua New Guinea, Sausi | 2012 | 3 | frequent | low | low low | high | RDT then LM | given separately | Peak of malaria in 0-4 year olds | 1132 |
| Rodriguez-Rodriguez [9] | Papua New Guinea, East Cape | 2013 | 2 | frequent | low | high low | low | RDT then LM | given separately | Peak of malaria in 0-4 year olds | 1187 |
| Rodriguez-Rodriguez [9] | Papua New Guinea, Karimui | 2011 | 2 | frequent | low | low low | high | RDT then LM | given separately | Peak of malaria in 0-4 year olds | 325 |
| Mitja [21] | Papua New Guinea, Lihir island | 2010 | 24 | frequent | low | low low | high | LM | not reported | Median age of people with case: 20 years | 9793 |
| Ome-Kaius [1], Lin [2], Betuela [3] | Papua New Guinea, Ilahita area | 2008 | 2 | frequent | low | high high | missing | LM | not reported | Study population under 55 months | 397 |
| Loha [19] | Ethiopia, Chano Mille | 2010 | 20 | long | low | low low | high | LM | not reported | 14.2% *Pf* and 23.2% *Pv* of episodes in under 5 year olds | 549 |
| Chaves^1^ [23], Kaneko [24] | Vanuatu | 1995 | 5 | frequent | low | low low | low | LM | not reported | Peak of malaria in 3-4 year olds (*Pv* and *Pf*) | 34372 |
| Sena [30] | Ethiopia, gilgel gibe hydroelectric dam | 2009 | 3 | long | low | low low | missing | missing | not reported | 10.5% of cases in under 5-year-olds | 12651 |
| Sena [30] | Ethiopia, gilgel gibe hydroelectric dam, control area | 2009 | 3 | long | low | low low | missing | missing | not reported | 12.7% of cases in under 5-year-olds | 22268 |

*^1^data added as yearly average*

Table D Characteristics of series of time-points: first-time MDA clinical cases

| **First author** | **Study area** | **Year of baseline** | **Number of data points** | **Relapse pattern** | **Sea-son-ality** | **Trans-**  **mission *Pf* *Pv*** | **Coverage** | **Diag-nostic tool** | **Mixed cases** | **Age groups** | **Total number of cases found in series of time-point** |
| --- | --- | --- | --- | --- | --- | --- | --- | --- | --- | --- | --- |
| Garfield^1^ [15] | Nicaragua | 1981 | 17 | long | low | low low | low | LM | not reported | Not reported | 26100 |
| McGreevy^2^ [29] | Brazil, Rondonia, costa Margue + Forte principe de beira + settlement along BR429 | 1986 | 13 | both | low | high high | missing | LM | not reported | 13-18% of population under 5 years, 45-50% under 14 years | 18617 |
| Kondrashin^3^ [40] | India, Andhra Pradesh, Donubai | 1981 | 8 | both | high | low low | low | LM | not reported | Not reported | 165 |
| Kondrashin^3^ [40] | India, Andhra Pradesh, Mondemkalu | 1981 | 8 | both | high | low low | low | LM | not reported | Not reported | 107 |
| Kondrashin^3^ [40] | India, Andhra Pradesh, Anantagiri | 1981 | 6 | both | high | low low | low | LM | not reported | Not reported | 121 |
| Kondrashin^3^ [40] | India, Andhra Pradesh, Dumbriguda | 1981 | 5 | both | high | low low | low | LM | not reported | Not reported | 41 |
| Kondrashin^3^ [40] | India, Andhra Pradesh, Downuru | 1981 | 12 | both | high | low low | low | LM | not reported | Not reported | 1172 |
| Kondrashin^3^ [40] | India, Andhra Pradesh, Thimmamapali | 1981 | 12 | both | high | low low | low | LM | not reported | Not reported | 102 |
| Kondrashin^3^ [40] | India, Andhra Pradesh, Atmakur | 1981 | 12 | both | high | low low | low | LM | not reported | Not reported | 281 |
| Kondrashin^3^ [40] | India, Andhra Pradesh, Palakur | 1981 | 12 | both | high | low low | low | LM | not reported | Not reported | 297 |
| Landier^4^ [50] | Myanmar, Kayin state | 2015 | 34 | frequent | high | low high | high | RDT | not reported | Not reported | 8204 |
| Chaumeau [7], Landier [8] | Myanmar, Kayin State, B2-HKT | 2014 | 20 | frequent | high | high high | low | RDT or LM | counted towards both | Median age of population 19 | 185 |
| Chaumeau [7], Landier [8] | Myanmar, Kayin State, B1-TPN | 2014 | 20 | frequent | high | low high | high | RDT or LM | counted towards both | Median age of population 19 | 44 |

*^1^time-point before intervention was implemented is an average over several years, this is a national study, however, coverage of large part of population (around 70%) within a month ^2^migration and resistance of P. falciparum to the drug ^3^some movement of workers into this area from elsewhere ^4^hotspot villages where the villages with higher malaria prevalence, the different villages could receive MDA at different times, results are all hot spot villages taken together with time-point zero when MDA was implemented, due to different time-points of MDA, follow-up times could vary which means data longer after time-point zero would consist of fewer villages, therefore the sample size would be smaller, this could not be taken account of in the analysis as it was assumed that the number of people at risk would remain the same throughout*

Table E Characteristics of series of time-points: repeated time MDA clinical cases

| **First author** | **Study area** | **Year of baseline** | **Number of data points** | **Relapse pattern** | **Sea-son-**  **ality** | **Trans-mission *Pf* *Pv*** | **Coverage** | **Diag-nostic tool** | **Mixed cases** | **Age groups** | **Total number of cases found in series of time-point** |
| --- | --- | --- | --- | --- | --- | --- | --- | --- | --- | --- | --- |
| Kondrashin^1^ [40] | India, Andhra Pradesh, Donubai | 1981 | 10 | both | high | low low | low | LM | not reported | Not reported | 41 |
| Kondrashin^1^ [40] | India, Andhra Pradesh, Mondemkalu | 1981 | 10 | both | high | low low | low | LM | not reported | Not reported | 43 |
| Kondrashin^1^ [40] | India, Andhra Pradesh, Anantagiri | 1981 | 12 | both | high | low low | low | LM | not reported | Not reported | 386 |
| Kondrashin^1^ [40] | India, Andhra Pradesh, Dumbriguda | 1981 | 12 | both | high | low low | low | LM | not reported | Not reported | 17 |

*^1^some movement of workers into this area from elsewhere*

Table F Characteristics of series of time-points: IRS clinical cases

| **First author** | **Study area** | **Year of baseline** | **Number of data points** | **Relapse pattern** | **Sea-son-ality** | **Trans-mission *Pf* *Pv*** | **Coverage** | **Diag-nostic tool** | **Mixed cases** | **Age groups** | **Total number of cases found in series of time-point** |
| --- | --- | --- | --- | --- | --- | --- | --- | --- | --- | --- | --- |
| Loha [19] | Ethiopia, Chano Mille | 2009 | 12 | long | low | low low | high | LM | not reported | 14.2% of *Pf* cases in under 5-year-olds, 23.2% of *Pv* cases in under 5 | 327 |
| Gabaldon^1^ [25] | Venezuela, eastern | 1950 | 2 | both | low | low low | missing | LM | mixed given but could be *Pm* too, hence not added | Peak of malaria in 1-4-year-olds | 487 |
| Gabaldon^1^ [25] | Venezuela, western | 1950 | 5 | both | low | low low | missing | LM | mixed given but could be *Pm* too, hence not added | Peak of malaria in 1-4-year-olds | 5201 |
| Kattenberg^2^ [26] | Vietnam, Nam Tra My District | 2013 | 2 | frequent | high | low low | missing | missing | not reported | 21-24% of participants under 5 | 565 |
| Charlwood^3^ [28] | Brazil, Rondonia, Machadinho | 1987 | 4 | both | low | high high | high | LM | not reported | Not reported | 38316 |
| Charlwood^3^ [28] | Brazil, Rondonia, Jaru | 1987 | 4 | both | low | high high | high | LM | not reported | Not reported | 42181 |
| Loha [34] | Ethiopia, Adami Tullu | 2015 | 2 | long | low | low low | high | RDT | given separately | 18.4% of population under 5 | 721 |
| Doke^4^ [35] | India, Akola District | 1997 | 4 | both | high | low low | high | LM | not reported | Not reported | 82 |
| Mishra [43] | India, Balaghat district | 2013 | 12 | both | high | low low | missing | LM | not reported | 25% of cases in under 5 | 410 |
| Rowland^5^ [45] | Pakistan | 1994 | 15 | long | low | low low | high | LM | not reported | Not reported | 4912 |
| Rowland^5^ [45] | Pakistan | 1994 | 12 | long | low | low low | high | LM | not reported | Not reported | 9682 |
| Rowland^6^ [46] | Pakistan, Sheikhupura district | 1997 | 2 | long | high | low high | high | LM | not reported | Not reported | 209 |
| Rowland^6^ [46] | Pakistan, Sheikhupura district | 1997 | 2 | long | high | low high | high | LM | not reported | Not reported | 204 |
| Seyoum [54] | Ethiopia, jimma zone, gilgel-gibe hydroelectric power dam reservoir | 2009 | 18 | long | low | high low | missing | LM | not reported | Population includes children under 10, of which 47% under 3 years | 681 |
| Singh [53] | India, Betul district | 2001 | 5 | both | high | high low | high | LM | counted towards *falciparum* | Below 10 year olds | 293 |
| Singh [53] | India, Betul district | 2001 | 5 | both | high | high low | high | LM | counted towards *falciparum* | Above 10 year olds | 540 |

*^1^Did not only test fever patients but some healthy people too ^2^data on district level was used and not commune level to have less stochasticity, judged on diagrams of rainfall May was considered wet season ^3^migration in this area ^4^spraying after peak of P. vivax. There was spraying before but due to resistance, used lambdacyhalothrin instead of DDT additionally to react to increasing numbers of malaria ^5^villages combined because of few case numbers. Spraying during different time-points of year: April in some villages and July in others, only villages with same month of spraying were combined, study area in a refugee settlement therefore could be influenced by migration ^6^data of different villages combined*

### **Characteristics of the series of time-points: patent infections**

Table G Characteristics of series of time-points: first-time ITN patent infections

| **First author** | **Study area** | **Year of baseline** | **Number of data points** | **Relapse pattern** | **Sea-son-ality** | **Trans-mission *Pf* *Pv*** | **Coverage** | **Diag-nostic tool** | **Mixed cases** | **Age groups** | **Total number of cases found in series of time-point** |
| --- | --- | --- | --- | --- | --- | --- | --- | --- | --- | --- | --- |
| Ome-Kaius [1], Lin [2], Betuela [3] | Papua New Guinea, Ilahita area | 2006 | 2 | frequent | low | high high | high | PCR and LM | not reported | Study population below 55 months | 339 |
| Koepfli [5] | Papua New Guinea, Madang area | 2006 | 2 | frequent | low | high high | missing | PCR and LM | counted towards both | 9-14% of study participants under 3 | 961 |
| Chaumeau [7], Landier [8] | Myanmar, Kayin State, B1-TPN | 2013 | 4 | frequent | high | low high | high | PCR | given separately | Median age of participants: 20 | 189 |
| Chaumeau [7],  Landier [8] | Myanmar, Kayin State, B2-HKT | 2013 | 4 | frequent | high | high high | high | PCR | given separately | Median age of participants 19 | 435 |
| Hetzel [14] | Papua New Guinea, Mumeng | 2009 | 2 | frequent | low | high high | low | LM | not reported | Median age 25 at baseline, at later time-point: 16 | 71 |
| Hetzel [14] | Papua New Guinea, Sausi | 2008 | 2 | frequent | low | low high | low | LM | not reported | Median age 18 at baseline, at later time-point: 19 | 54 |
| Hetzel [14] | Papua New Guinea, Tabibuga | 2009 | 2 | frequent | low | high low | low | LM | not reported | Median age 27 at baseline, at later time-point: 25 | 40 |
| Hetzel [14] | Papua New Guinea, Yapsie | 2009 | 2 | frequent | low | low low | high | LM | not reported | Median age at baseline: 18, at later time-point: 19 | 190 |
| Hetzel [14] | Papua New Guinea, Finschhafen | 2009 | 2 | frequent | low | low low | low | LM | not reported | Median age at baseline: 19, at later time-point: 16 | 57 |
| Thomson^1^ [33] | Cambodia, stung treng, mondul kiri, kampong thom, kampong cham, plantation workers | 2014 | 2 | frequent | high | low low | low | PCR | given separately | 50% 15-30 years old, 50% above 30 years old | 40 |
| Rowland^2^ [39] | Afghanistan | 1995 | 4 | long | low | low high | low | LM | not reported | 21% of population 0-4-year-olds, mean age 18.3 | 340 |
| Rowland^2^ [39] | Afghanistan | 1996 | 2 | long | low | low high | low | LM | not reported | 21% of population 0-4-year-olds, mean age 16.9 | 174 |
| Smithuis^3^ [49] | Myanmar, rakhine state, sittwe and maungdaw | 1997 | 4 | frequent | high | high high | high | LM | counted towards both | Study population: Children under 10 | 1741 |
| Hii^4^ [38] | Solomon Islands | 1988 | 2 | frequent | low | high high | high | LM | not reported | 0-9-year-olds | 24 |
| Rowland [51], Rowland [52] | Pakistan, Mardan district, Baghicha and Kagan | 1991 | 6 | long | low | low high | high | LM | not reported | Mean age of population 19 | 645 |

*^1^population includes many temporary workers that have been in area less than 6 months ^2^only parts of the community received nets, number of infected people found is given separately for people with nets and people without, for the analysis these numbers were added to give a community effect ^3^data of villages combined due to few case numbers ^4^combination of the different regions because of few infected people in individual areas*

Table H Characteristics of series of time-points: repeated time ITN patent infections

| **First author** | **Study area** | **Year of baseline** | **Number of data points** | **Relapse pattern** | **Sea-son-ality** | **Trans-mission *Pf* *Pv*** | **Coverage** | **Diag-nostic tool** | **Mixed cases** | **Age groups** | **Total number of cases found in series of time-point** |
| --- | --- | --- | --- | --- | --- | --- | --- | --- | --- | --- | --- |
| Deressa [10] | Ethiopia, SNNPR, Halaba woreda district | 2008 | 3 | long | low | low low | high | LM | given separately | Mean age of population: 19, 15.5% under 5-year-olds | 68 |
| Hetzel^1^ [11-13] | Papua New Guinea, National Capital District | 2011 | 3 | frequent | low | low low | low | LM | counted towards both | 10-15% of surveyed people under 5 | 5 |
| Hetzel^1^ [11-13] | Papua New Guinea, Milne Bay | 2011 | 3 | frequent | low | low low | high | LM | counted towards both | 10-15% of surveyed people under 5 | 102 |
| Hetzel^1^ [11-13] | Papua New Guinea, Southern Highlands | 2011 | 2 | frequent | low | low low | high | LM | counted towards both | 10-15% of surveyed people under 5 | 6 |
| Hetzel^1^ [11-13] | Papua New Guinea, Eastern Highlands | 2009 | 3 | frequent | low | low low | low | LM | counted towards both | 10-15% of surveyed people under 5 | 10 |
| Hetzel^1^ [11-13] | Papua New Guinea, Morobe | 2011 | 2 | frequent | low | low low | low | LM | counted towards both | 10-15% of surveyed people under 5 | 33 |
| Hetzel^1^ [11-13] | Papua New Guinea, Sandaun | 2014 | 2 | frequent | low | low low | low | LM | counted towards both | 10-15% of surveyed people under 5 | 72 |
| Hetzel^1^ [11-13] | Papua New Guinea, Manus | 2014 | 2 | frequent | low | low low | high | LM | counted towards both | 10-15% of surveyed people under 5 | 9 |
| Hetzel^1^ [11-13] | Papua New Guinea, New Ireland | 2014 | 2 | frequent | low | low low | high | LM | counted towards both | 10-15% of surveyed people under 5 | 65 |
| Hetzel^1^ [11-13] | Papua New Guinea, East new Britain | 2014 | 2 | frequent | low | low low | high | LM | counted towards both | 10-15% of surveyed people under 5 | 46 |
| Hetzel^1^ [11-13] | Papua New Guinea, Morobe | 2014 | 2 | frequent | low | low low | high | LM | counted towards both | 10-15% of surveyed people under 5 | 29 |
| Luxemburger [16] | Thai-myanmar border, shoklo camp | 1990 | 3 | frequent | high | high low | high | LM | not reported | 4-15 year old children | 41 |
| Sluydts^2^ [47], Sluydts [48] | Cambodia, Ratanakiri province | 2012 | 5 | frequent | high | low low | high | PCR | not reported | Median age 18, peak of malaria in 5-14 year olds | 838 |
| Ome-Kaius [1], Lin [2], Betuela [3] | Papua New Guinea, Ilahita area | 2008 | 2 | frequent | low | high high | missing | PCR and LM | not reported | Study population below 55 months | 205 |
| Koepfli [5] | Papua New Guinea, Madang area | 2010 | 2 | frequent | low | high high | missing | PCR and LM | counted towards both | 9-14% of study participants under 3 | 440 |
| Kattenberg [4] | Papua New Guinea, Ilahita 3 | 2005 | 2 | frequent | low | high low | high | PCR | counted towards both | Median age at baseline 17, at later time-point: 22 | 148 |
| Kattenberg [4] | Papua New Guinea, Ilahita 4 | 2005 | 2 | frequent | low | high low | high | PCR | counted towards both | Median age at baseline 17, at later time-point: 22 | 171 |
| Kattenberg [4] | Papua New Guinea, Sunuhu | 2005 | 2 | frequent | low | high low | high | PCR | counted towards both | Median age at baseline 17, at later time-point: 22 | 395 |
| Kattenberg [4] | Papua New Guinea, Urita | 2005 | 2 | frequent | low | high low | high | PCR | counted towards both | Median age at baseline 17, at later time-point: 22 | 204 |
| Kattenberg [4] | Papua New Guinea, Waragom | 2005 | 2 | frequent | low | high low | high | PCR | counted towards both | Median age at baseline 17, at later time-point: 22 | 218 |
| Kattenberg [4] | Papua New Guinea, Jama | 2005 | 2 | frequent | low | high low | high | PCR | counted towards both | Median age at baseline 17, at later time-point: 22 | 147 |
| Kattenberg [4] | Papua New Guinea, Sengo | 2005 | 2 | frequent | low | high low | high | PCR | counted towards both | Median age at baseline 17, at later time-point: 22 | 158 |
| Kattenberg [4] | Papua New Guinea, Maiwi | 2005 | 2 | frequent | low | high low | high | PCR | counted towards both | Median age at baseline 17, at later time-point: 22 | 123 |
| Kattenberg [4] | Papua New Guinea, Malba 1 | 2005 | 2 | frequent | low | high low | high | PCR | counted towards both | Median age at baseline 17, at later time-point: 22 | 161 |
| Kattenberg [4] | Papua New Guinea, Malba 2 | 2005 | 2 | frequent | low | high low | high | PCR | counted towards both | Median age at baseline 17, at later time-point: 22 | 184 |
| Kattenberg [4] | Papua New Guinea, Wombisa | 2005 | 2 | frequent | low | high low | high | PCR | counted towards both | Median age at baseline 17, at later time-point: 22 | 157 |

*^1^distribution time-points of nets from Manuel Hetzel (personal communication) [56]. To calculate coverage of nets census data [57] was used. ^2^in baseline only looked at people above 2 years old, assumed this was the case for follow-up study as well. Four villages not accessible in follow up.*

Table I Characteristics of series of time-points: first-time MDA patent infections

| **First author** | **Study area** | **Year of baseline** | **Number of data points** | **Relapse pattern** | **Sea-son-ality** | **Trans-mission *Pf* *Pv*** | **Coverage** | **Diag-nostic tool** | **Mixed cases** | **Age groups** | **Total number of cases found in series of time-point** |
| --- | --- | --- | --- | --- | --- | --- | --- | --- | --- | --- | --- |
| McGreevy^1^ [29] | Brazil, Rondonia, settlement along BR429 | 1986 | 4 | both | low | high high | missing | LM | not reported | 14% of population under 5 | 352 |
| Hofmann [31], Robinson [32] | Papua New Guinea, East Sepik Province, Maprik district | 2009 | 13 | frequent | low | high high | high | LM | not reported | Study population 5-10-year-olds, mean age 7.6 years | 1030 |
| Hofmann [31], Robinson [32] | Papua New Guinea, East Sepik Province, Maprik district | 2009 | 13 | frequent | low | high high | high | LM | not reported | Study population 5-10-year-olds only, mean age 7.6 years | 587 |
| Kligler [36] | Palestine, Jahuleh | 1930 | 2 | long | high | high high | low | LM | not reported | 0-14-year-olds | 86 |
| Kligler [36] | Palestine, Melaha | 1930 | 2 | long | high | high low | low | LM | not reported | 0-14-year-olds | 128 |
| Kligler [36] | Palestine, Almanieh | 1930 | 2 | long | high | high high | low | LM | not reported | 0-14-year-olds | 25 |
| Van Dijk^2^ [37] | Indonesia, West-Papaua, Inanwatan | 1960 | 2 | frequent | low | high high | high | LM | counted towards both | 12-19% of population under 5 | 254 |
| Lwin^3^ [55] | Thai-Myanmar border | 2012 | 4 | frequent | high | high high | low | LM | counted towards both | Study population over 5 years of age only | 240 |
| Chaumeau [7] Landier [8] | Myanmar, Kayin State, B1-TPN | 2014 | 7 | frequent | high | low high | high | PCR | given separately | Median age of population 20 | 149 |
| Chaumeau [7], Landier [8] | Myanmar, Kayin State, B2-HKT | 2014 | 6 | frequent | high | low high | low | PCR | given separately | Median age of population 19 | 360 |

*^1^migraton and resistance of falciparum to chloroquine ^2^mode of detection assumed to be LM ^3^infections in children and adults combined due to low numbers*

Table J Characteristics of series of time-points: IRS patent infections

| **First author** | **Study area** | **Year of baseline** | **Number of data points** | **Relapse pattern** | **Season-ality** | **Trans-mission *Pf* *Pv*** | **Coverage** | **Diag-nostic tool** | **Mixed cases** | **Age groups** | **Total number of cases found in series of time-point** |
| --- | --- | --- | --- | --- | --- | --- | --- | --- | --- | --- | --- |
| Metselaar^1^ [17] | Indonesia, Papua, Sentani | 1953 | 5 | frequent | low | high high | missing | LM | not reported | Highest burden of malaria in 1 year olds | 1780 |
| Sharma [22] | India, Uttar Pradesh, Bareilly district | 2001 | 3 | long | high | low low | high | LM | not reported | Not reported | 207 |
| Hii^2^ [38] | Solomon Islands | 1988 | 2 | frequent | low | high high | high | LM | not reported | 0-9-year-olds | 43 |
| Rowland^3^ [44] | Pakistan, M. Khoja | 1993 | 2 | long | high | low high | high | LM | not reported | 5-15-year-olds | 42 |
| Rowland^3^ [44] | Pakistan, Darsamund | 1993 | 2 | long | high | low high | high | LM | not reported | 5-15-year-olds | 45 |
| Rowland^3^ [44] | Pakistan, Y.Ghund | 1993 | 2 | long | high | low high | high | LM | not reported | 5-15-year-olds | 48 |
| Rowland^3^ [44] | Pakistan, Azakhel | 1993 | 2 | long | high | low high | high | LM | not reported | 5-15-year-olds | 41 |
| Rowland^3^ [44] | Pakistan, Kotki | 1993 | 2 | long | high | low low | high | LM | not reported | 5-15-year-olds | 11 |
| Rowland [45] | Pakistan, Darsamund | 1994 | 2 | long | low | low high | high | LM | not reported | School children | 39 |
| Rowland [45] | Pakistan, Baghbanan | 1994 | 2 | long | low | low high | high | LM | not reported | School children | 43 |
| Rowland [45] | Pakistan, Badaber | 1994 | 2 | long | low | low high | high | LM | not reported | School children | 24 |
| Rowland [45] | Pakistan, Dallan | 1994 | 2 | long | low | low low | high | LM | not reported | School children | 15 |
| Rowland [45] | Pakistan, Khazana | 1994 | 2 | long | low | low low | high | LM | not reported | School children | 8 |
| Rowland [45] | Pakistan, Yakkaghund | 1994 | 2 | long | low | low low | high | LM | not reported | School children | 8 |
| Rowland [45] | Pakistan, Naguman | 1994 | 2 | long | low | low low | high | LM | not reported | School children | 20 |
| Rowland [45] | Pakistan, Chakdara | 1994 | 2 | long | low | low high | high | LM | not reported | School children | 30 |
| Rowland^4^ [46] | Pakistan, Sheikhupura district | 1997 | 2 | long | high | low high | high | LM | not reported | 5-15-year-olds | 107 |
| Rowland^4^ [46] | Pakistan, Sheikhupura district | 1997 | 2 | long | high | low high | high | LM | not reported | 5-15-year-olds | 123 |

^1^diagnostic method assumed to be LM, number of people tested only given for one survey, assumed to be the same for the others ^2^regions combined because of few infected people ^3^villages kept separate despite few numbers of infected people because they were purposely chosen from a range of different areas ^4^data of villages combined

**References**

1. Ome-Kaius M, Kattenberg JH, Zaloumis S, Siba M, Kiniboro B, Jally S, *et al*. Differential impact of malaria control interventions on *P. falciparum* and *P. vivax* infections in young Papua New Guinean children. *BMC Med* 2019;17(1):220.
2. Lin E, Kiniboro B, Gray L, Dobbie S, Robinson L, Laumaea A, et al. Differential patterns of infection and disease with *P. falciparum* and *P. vivax* in young Papua New Guinean children. *PLOS ONE* 2010;5(2):e9047.
3. Betuela I, Rosanas-Urgell A, Kiniboro B, Stanisic DI, Samol L, de Lazzari E, *et al*. Relapses contribute significantly to the risk of *Plasmodium vivax* infection and disease in Papua New Guinean children 1-5 years of age. *J Inf Dis* 2012;206(11):1771-80.
4. Kattenberg JH, Gumal DL, Ome-Kaius M, Kiniboro B, Philip M, Jally S, *et al*. The epidemiology of *Plasmodium falciparum* and *Plasmodium vivax* in East Sepik Province, Papua New Guinea, pre- and post-implementation of national malaria control efforts. *Malar J* 2020;19(1):198.
5. Koepfli C, Ome-Kaius M, Jally S, Malau E, Maripal S, Ginny J, *et al.* Sustained malaria control over an 8-year period in Papua New Guinea: The challenge of low-density asymptomatic Plasmodium infections. *J Inf Dis* 2017;216(11):1434-43.
6. Graves PM, Brabin BJ, Charlwood JD, Burkot TR, Cattani JA, Ginny M, *et al*. Reduction in incidence and prevalence of *Plasmodium falciparum* in under-5-year-old children by permethrin impregnation of mosquito nets. *Bull WHO* 1987;65(6):869-77.
7. Chaumeau V, Kajeechiwa L, Fustec B, Landier J, Naw Nyo S, Nay Hsel S, *et al.* Contribution of asymptomatic Plasmodium infections to the transmission of malaria in Kayin State, Myanmar. *J Inf Dis* 2019;219(9):1499-509.
8. Landier J, Kajeechiwa L, Thwin MM, Parker DM, Chaumeau V, Wiladphaingern J, *et al*. Safety and effectiveness of mass drug administration to accelerate elimination of artemisinin-resistant falciparum malaria: A pilot trial in four villages of Eastern Myanmar. *Wellcome Open Res*. 2017;2:81.
9. Rodriguez-Rodriguez D, Maraga S, Lorry L, Robinson LJ, Siba PM, Mueller I, et al. Repeated mosquito net distributions, improved treatment, and trends in malaria cases in sentinel health facilities in Papua New Guinea. *Malar J* 2019;18(1):364.
10. Deressa W, Yihdego YY, Kebede Z, Batisso E, Tekalegne A, Dagne GA. Effect of combining mosquito repellent and insecticide treated net on malaria prevalence in Southern Ethiopia: a cluster-randomised trial. *Parasit Vectors* 2014;7:132.
11. Hetzel MW, Pulford J, Ura Y, Jamea-Maiasa S, Tandrapah A, Tarongka N, *et al*. Insecticide-treated nets and malaria prevalence, Papua New Guinea, 2008-2014. *Bull WHO* 2017;95(10):695-705b.
12. Hetzel MW, Morris H, Tarongka N, Barnadas C, Pulford J, Makita L, *et al*. Prevalence of malaria across Papua New Guinea after initial roll-out of insecticide-treated mosquito nets. *Trop Med Int Health* 2015;20(12):1745-55.
13. Hetzel MW, Saweri OP, Kuadima JJ, Smith I, Ura Y, Tandrapah A, *et al.* Papua New Guinea malaria indicator survey 2016-2017: malaria prevention, infection and treatment. Papua New Guinea Institue of Medical Research, Goroka, 2018; 2018.
14. Hetzel MW, Reimer LJ, Gideon G, Koimbu G, Barnadas C, Makita L, *et al.* Changes in malaria burden and transmission in sentinel sites after the roll-out of long-lasting insecticidal nets in Papua New Guinea. *Parasit Vectors* 2016;9(1):340.
15. Garfield RM, Vermund SH. Changes in malaria incidence after mass drug administration in Nicaragua. *Lancet* 1983;2(8348):500-3.
16. Luxemburger C, Perea WA, Delmas G, Pruja C, Pecoul B, Moren A. Permethrin-impregnated bed nets for the prevention of malaria in schoolchildren on the Thai-Burmese border. *Trans Roy Soc Trop Med Hyg* 1994;88(2):155-9.
17. Metselaar D. Seven years' malaria research and residual house spraying in Netherlands New Guinea. *Am J Trop Med Hyg* 1961;10:327-34.
18. Kessler A, van Eijk AM, Jamir L, Walton C, Carlton JM, Albert S. Malaria in Meghalaya: a systematic literature review and analysis of data from the National Vector-Borne Disease Control Programme. *Malar J* 2018;17(1):411.
19. Loha E, Lunde TM, Lindtjørn B. Effect of bednets and indoor residual spraying on spatio-temporal clustering of malaria in a village in south Ethiopia: a longitudinal study. *PLOS ONE* 2012;7(10):e47354.
20. Hanafi-Bojd AA, Vatandoost H, Oshaghi MA, Haghdoost AA, Shahi M, Sedaghat MM, et al. Entomological and epidemiological attributes for malaria transmission and implementation of vector control in southern Iran. Acta tropica. 2012;121(2):85-92.
21. Mitjà O, Paru R, Selve B, Betuela I, Siba P, De Lazzari E, *et al.* Malaria epidemiology in Lihir Island, Papua New Guinea. *Malar J* 2013;12:98.
22. Sharma SN, Shukla RP, Raghavendra K, Subbarao SK. Impact of DDT spraying on malaria transmission in Bareilly District, Uttar Pradesh, India. *J Vector Borne Dis* 2005;42(2):54-60.
23. Chaves LF, Kaneko A, Taleo G, Pascual M, Wilson ML. Malaria transmission pattern resilience to climatic variability is mediated by insecticide-treated nets. *Malar J* 2008;7:100.
24. Kaneko A, Taleo G, Kalkoa M, Yaviong J, Reeve PA, Ganczakowski M, *et al*. Malaria epidemiology, glucose 6-phosphate dehydrogenase deficiency and human settlement in the Vanuatu Archipelago. *Acta Trop* 1998;70(3):285-302.
25. Gabaldon A, Guerrero L. An attempt to eradicate malaria by the weekly administration of pyrimethamine in areas of out-of-doors transmission in Venezuela. *Am J Trop Med Hyg* 1959;8(4):433-9.
26. Kattenberg JH, Erhart A, Truong MH, Rovira-Vallbona E, Vu KAD, Nguyen THN, *et al*. Characterization of *Plasmodium falciparum* and *Plasmodium vivax* recent exposure in an area of significantly decreased transmission intensity in Central Vietnam. *Malar J* 2018;17(1):180.
27. Maude RJ, Nguon C, Ly P, Bunkea T, Ngor P, Canavati de la Torre SE, *et al*. Spatial and temporal epidemiology of clinical malaria in Cambodia 2004-2013. *Malar J* 2014;13:385.
28. Charlwood JD, Alecrim WD, Fe N, Mangabeira J, Martins VJ. A field trial with lambda-cyhalothrin (ICON) for the intradomiciliary control of malaria transmitted by Anopheles darlingi root in Rondonia, Brazil. *Acta Trop* 1995;60(1):3-13.
29. McGreevy PB, Dietze R, Prata A, Hembree SC. Effects of immigration on the prevalence of malaria in rural areas of the Amazon basin of Brazil. *Memorias do Instituto Oswaldo Cruz* 1989;84(4):485-91.
30. Sena LD, Deressa WA, Ali AA. Analysis of trend of malaria prevalence in south-west Ethiopia: a retrospective comparative study. *Malar J* 2014;13:188.
31. Hofmann NE, Karl S, Wampfler R, Kiniboro B, Teliki A, Iga J *et al*. The complex relationship of exposure to new Plasmodium infections and incidence of clinical malaria in Papua New Guinea. *eLife* 2017;6.
32. Robinson LJ, Wampfler R, Betuela I, Karl S, White MT, Li Wai Suen CS *et al.* Strategies for understanding and reducing the *Plasmodium vivax* and *Plasmodium ovale* hypnozoite reservoir in Papua New Guinean children: a randomised placebo-controlled trial and mathematical model. *PLOS Med* 2015;12(10):e1001891.
33. Thomson R, Sochea P, Sarath M, MacDonald A, Pratt A, Poyer S*, et al.* Rubber plantations and drug resistant malaria: a cross-sectional survey in Cambodia. *Malar J* 2019;18(1):379.
34. Loha E, Deressa W, Gari T, Balkew M, Kenea O, Solomon T *et al*. Long-lasting insecticidal nets and indoor residual spraying may not be sufficient to eliminate malaria in a low malaria incidence area: results from a cluster randomized controlled trial in Ethiopia. *Malar J* 2019;18(1):141.
35. Doke PP, Sathe RS, Chouhan SP, Bhosale AS. Impact of single round of indoor residual spray with lambda-cyhalotrin 10% WP on Plasmodium falciparum infection in Akola district, Maharashtra State. *J Communic Dis* 2000;32(3):190-200.
36. Kligler I, Mer G. Periodic intermittent treatment with chinoplasmine as a measure of malaria control in a hyperendemic area. *Revista di Malariologia* 1931;10(4).
37. van DW. Mass treatment of malaria with chloroquine: Results of a trial in Inanwatan. *Trop Geogr Med* 1961;13:351-6.
38. Hii JL, Kanai L, Foligela A, Kan SK, Burkot TR, Wirtz RA. Impact of permethrin-impregnated mosquito nets compared with DDT house-spraying against malaria transmission by *Anopheles farauti* and *An.punctulatus* in the Solomon Islands. *Med Vet Entomol* 1993;7(4):333-8.
39. Rowland M, Webster J, Saleh P, Chandramohan D, Freeman T, Pearcy B *et al*. Prevention of malaria in Afghanistan through social marketing of insecticide-treated nets: evaluation of coverage and effectiveness by cross-sectional surveys and passive surveillance. *Trop Med Int Health* 2002;7(10):813-22.
40. Kondrashin AV, Sanyal MC. Mass drug administration in Andhra Pradesh in areas under *Plasmodium falciparum* containment programme. *J Communic Dis* 1985;17(4):293-9.
41. Sahu SS, Jambulingam P, Vijayakumar T, Subramanian S, Kalyanasundaram M. Impact of alphacypermethrin treated bed nets on malaria in villages of Malkangiri district, Orissa, India. *Acta Trop* 2003;89(1):55-66.
42. Shah NK, Tyagi P, Sharma SK. The impact of artemisinin combination therapy and long-lasting insecticidal nets on forest malaria incidence in tribal villages of India, 2006-2011. *PLOS ONE* 2013;8(2):e56740.
43. Mishra AK, Bharti PK, Kareemi TI, Chand SK, Tidgam AS, Sharma RK *et al.* Field evaluation of zero vector durable lining to assess its efficacy against malaria vectors and malaria transmission in tribal areas of the Balaghat district of central India. *Trans Roy Soc Trop Med Hyg* 2019;113(10):623-31.
44. Rowland M, Hewitt S, Durrani N. Prevalence of malaria in Afghan refugee villages in Pakistan sprayed with lambdacyhalothrin or malathion. *Trans Roy Soc Trop Med Hyg* 1994;88(4):378-9.
45. Rowland M, Hewitt S, Durrani N, Bano N, Wirtz R. Transmission and control of vivax malaria in Afghan refugee settlements in Pakistan. *Trans Roy Soc Trop Med Hyg* 1997;91(3):252-5.
46. Rowland M, Mahmood P, Iqbal J, Carneiro I, Chavasse D. Indoor residual spraying with alphacypermethrin controls malaria in Pakistan: a community-randomized trial. *Trop Med Int Health* 2000;5(7):472-81.
47. Sluydts V, Durnez L, Heng S, Gryseels C, Canier L, Kim S *et al.* Efficacy of topical mosquito repellent (picaridin) plus long-lasting insecticidal nets versus long-lasting insecticidal nets alone for control of malaria: a cluster randomised controlled trial. *Lancet Inf Dis 2*016;16(10):1169-77.
48. Sluydts V, Heng S, Coosemans M, Van Roey K, Gryseels C, Canier L *et al*. Spatial clustering and risk factors of malaria infections in Ratanakiri Province, Cambodia. *Malar J* 2014;13:387.
49. Smithuis FM, Kyaw MK, Phe UO, van der Broek I, Katterman N, Rogers C *et al*. The effect of insecticide-treated bed nets on the incidence and prevalence of malaria in children in an area of unstable seasonal transmission in western Myanmar. *Malar J* 2013;12:363.
50. Landier J, Parker DM, Thu AM, Lwin KM, Delmas G, Nosten FH. Effect of generalised access to early diagnosis and treatment and targeted mass drug administration on *Plasmodium falciparum* malaria in Eastern Myanmar: an observational study of a regional elimination programme. *Lancet* 2018;391(10133):1916-26.
51. Rowland M, Bouma M, Ducornez D, Durrani N, Rozendaal J, Schapira A, et al. Pyrethroid-impregnated bed nets for personal protection against malaria for Afghan refugees. *Trans Roy Soc Trop Med Hyg* 1996;90(4):357-61.
52. Rowland M, Hewitt S, Durrani N, Saleh P, Bouma M, Sondorp E. Sustainability of pyrethroid-impregnated bednets for malaria control in Afghan communities. *Bull WHO* 1997;75(1):23-9.
53. Singh N, Shukla MM, Mishra AK, Singh MP, Paliwal JC, Dash AP. Malaria control using indoor residual spraying and larvivorous fish: a case study in Betul, central India. *Trop Med Int Health* 2006;11(10):1512-20.
54. Seyoum D, Kifle YG, Rondeau V, Yewhalaw D, Duchateau L, Rosas-Aguirre A, et al. Identification of different malaria patterns due to *Plasmodium falciparum* and *Plasmodium vivax* in Ethiopian children: a prospective cohort study. *Malar J* 2016;15:208.
55. Lwin KM, Imwong M, Suangkanarat P, Jeeyapant A, Vihokhern B, Wongsaen K, et al. Elimination of *Plasmodium falciparum* in an area of multi-drug resistance. *Malar J* 2015;14:319.
56. Hetzel MW. Personal communication.
57. National Statistics Office. Papua New Guinea 2011 National Report - Census 2011. 2012.
