# Supplementary material for: The relative impact of interventions on sympatric Plasmodium vivax and Plasmodium falciparum malaria: A systematic review
Source: PLoS Negl Trop Dis. 2022 Jun 29;16(6):e0010541. doi: 10.1371/journal.pntd.0010541 (PMC9242512; doi:10.1371/journal.pntd.0010541)
Supplement: S2 Supporting Information — (DOCX) [file pntd.0010541.s002.docx]

**S2 Supporting information: Interventions in place at baseline**

Table A Case detection, baseline interventions, MDA regimen and insecticide used (where applicable) for studies with information on clinical cases

| **First time ITN distribution** | | | | |
| --- | --- | --- | --- | --- |
| **First author** | **Case detection** | **Baseline intervention*** | | |
| Ome-Kaius [1], Lin [2], Betuela [3] | Active and passive | Varying coverage of nets (0-98%) in different villages | | |
| Hetzel [4] | Passive | Some nets owned previously (usage 2-18%) | | |
| Kessler [5] | Passive | IRS and ITN (unknown coverage) before (IRS with low acceptance), some education, repellents and coils | | |
| Chaves [6], Kaneko [7] | Passive | IRS was done before ITNs were introduced but then abandoned | | |
| Loha [8] | Active and Passive | Used to have IRS, but no IRS the year prior to study, previous ITN coverage was around 11% | | |
| Shah [9] | Active and Passive | Spraying (coverage unknown) happening up until the ITNs were introduced, they changed to ACT in January 2010, around the time of the second data point | | |
| Rowland^1^ [10, 11] | Passive | Spraying was taken up during the study, a retreatment happened (both after first data point but before the second data points in figure 3j) which was not included in the analysis as it was more than 24 months after the intervention) | | |
| Sahu [12] | Active | Retreatment 3 months after distribution of nets | | |
| Maude [13] | Passive | VMW^2^ were there before, also some ITNs but low coverage | | |
| Sena [14] | Passive | IRS, around the same time health extension workers were used for diagnosis and treatment of uncomplicated cases | | |
| Chaumeau [15], Landier [16] | Passive | Increased the number of malaria posts at that time | | |
| **Repeated time ITN distribution** | | | | |
| **First author** | **Case detection** | **Baseline intervention*** | | |
| Rodriguez-Rodriguez [17] | Passive | Nothing specific mentioned | | |
| Mitja [18] | Passive | IRS done bi-annually, vector breeding site reduction once a week if anophelines present | | |
| Ome-Kaius [1], Lin [2], Betuela [3] | Active and passive | Nothing specific mentioned | | |
| Loha [19] | Active and passive | Spraying around 9 months before | | |
| Chaves [6], Kaneko [7] | Passive | Nothing specific mentioned | | |
| Sena [14] | Passive | Did IRS throughout the study but with a change from DDT and malathion to deltamethrin or bendiocarb around the same time as ITN distribution | | |
| **Mass drug administration** | | | | |
| **First author** | **Case detection** | **Baseline intervention*** | **MDA drugs** | **Number of monthly rounds** |
| Garfield [20] | Passive | Breeding site control campaign a month before MDA | Chloroquine (600mg/450mg/450mg), primaquine (15mg/15mg/15mg) | One time for three days |
| McGreevy [21] | Passive | Spraying with DDT and some irregular outdoor spraying | No information | Every month for three months |
| Kondrashin [22] | Passive | Spraying every year shortly before monsoon, figure 7n)-q) had previous round of MDA | Chloroquine (600mg), primaquine (45mg) | One time |
| Landier [23] | Passive | Increased early diagnosis at this time, could get ITNs if needed | Dihydroartemisinin (7mg/kg), piperaquine tetraphosphate (55mg/kg) | Three days every month for three months |
| Chaumeau [15], Landier [16] | Passive | Around 8 months before: malaria posts for early diagnosis and ITNs distributed | Dihydroartemisinin (7mg/kg), piperaquine tetraphosphate (55mg/kg), primaquine (0.25mg/kg) | Three days every month for three months |
| **Indoor residual spraying** | | | | |
| **First author** | **Case detection** | **Baseline intervention*** | | **Insecticide used** |
| Gabaldon^3^ [24] | Active | Some areas had IRS before | | DDT |
| Loha c) [8] | Active and passive | No IRS year prior to study, ITN coverage was around 11% | | Propoxur |
| Singh [25] | Active | Spraying in year before but with DDT, added some fish (not assumed to have a big impact), increased case management around 6 months before | | Pyrethroid |
| Seyoum [26] | Active and passive | Nothing specific mentioned, a net distribution started towards the end of the study | | Deltamethrin |
| Doke [27] | Passive | In previous years sprayed with DDT | | Lambdacyhalothrin |
| Kattenberg [28] | Passive | Nothing specific mentioned (but likely that there was control) | | Not reported |
| Charlwood [29] | Passive | Nothing specific mentioned | | DDT (1year) then lambdacyhalothrin |
| Mishra [30] | Active | Nothing specific mentioned | | Alphacypermethrin |
| Loha h) [19] | Active | Had some nets (coverage unknown) | | DDT |
| Rowland (m/n) [31] | Passive | Nothing specific mentioned | | Pyrethroid lambdacyhalothrin (5 villages), malathion (9 villages) |
| Rowland (o/p) [32] | Active | Nothing specific mentioned | | Alphacypermethrin |

*^1^ Only 10% of population received nets, clinical cases of people that received nets were compared to cases of people that did not receive nets*

*^2^ Village malaria workers: provided diagnosis and treatment*

*^3^ Also tested some healthy people*

**Most areas had some level of case management*

Table B Baseline interventions, MDA regimen and insecticide used (where applicable) for studies with information on patent infections

| **First time ITN distribution** | | | | |
| --- | --- | --- | --- | --- |
| **First author** | **Baseline intervention*** | | | |
| Ome-Kaius [1], Lin [2], Betuela [3] | Varying coverage of nets (0-98%) in different villages | | | |
| Koepfli [33] | Nothing specific mentioned | | | |
| Hetzel [4] | Nothing specific mentioned | | | |
| Thomson [34] | Plantation malaria workers for diagnosis/treatment and some distribution of ITNs | | | |
| Rowland (Afghanistan) [35] | 12% of families had nets before | | | |
| Smithuis [36] | Mainly early diagnosis and treatment | | | |
| Rowland^1^  (Pakistan) [10, 11] | Spraying was taken up during the study (between second and third measurement, however, not included in analysis because more than 24 months after ITN distribution and therefore not relevant for analysis), a retreatment (happened before second collection of prevalence data) | | | |
| Hii [37] | DDT spraying but stopped 18 months before, one retreatment happened before data point collection | | | |
| Chaumeau [15], Landier [16] | Increased the number of malaria posts at that time | | | |
| **Repeated time ITN distribution** | | | | |
| **First author** | **Baseline intervention*** | | | |
| Deressa^2^ [38] | At baseline: 65% had one ITN per household and 35% two per household | | | |
| Hetzel[39-41] | Nothing specific mentioned | | | |
| Luxemburger^3^ [42] | Nothing specific mentioned | | | |
| Sluydts [43, 44] | Nets with high coverage, promotion of nets | | | |
| Ome-Kaius [1], Lin [2], Betuela [3] | Nothing specific mentioned | | | |
| Koepfli [33] | Nothing specific mentioned | | | |
| Kattenberg [45] | Nothing specific mentioned. Coverage around 88% but mostly untreated nets | | | |
| **Mass drug administration** | | | | |
| **First author** | **Baseline intervention*** | **MDA drugs** | | **Number of monthly rounds** |
| McGreevy [21] | Spraying with DDT of houses, also some malathion spraying outdoors in town | Not recorded | | Not recorded |
| Hofmann^4^ [46], Robinson [47] | High coverage of ITN present | Artemeter-lumefantrine (over 3 days) and Chloroquine (over 3 days 25mg/kg total dose) and in 8c): Primaquine 20 days (0.5 mg/kg/day). In 8b): placebo | | One round, 20 days |
| Kligler [48] | None mentioned, there was a previous study with same procedure, not clear if in this area | Daily dosage adults for 5 days: plasmochine (30mg) and quinine sulphate (900mg), twice daily | | 3 rounds in total every 3 weeks |
| Van Dijk [49] | There was spraying before, but this was stopped for the study | Chloroquine if above 45 kg then a dose of 450mg (also diethylcarbamazine for filariasis) | | Eleven rounds, every 4 weeks measurement after 10 rounds |
| Lwin [50] | Nothing specific mentioned | (3 days of dihydroartemisinin-piperaquine 7.5/60mg/kg) only people above 14 years | | Three rounds in total, one each month |
| Chaumeau [15], Landier [16] | Around 8 months before: malaria posts for early diagnosis and ITNs distributed | Low dose primaquine, dihydroartemisinin-piperaquine | | Three rounds in total, one each month |
| **Indoor residual spraying** | | | | |
| **First author** | **Baseline intervention*** | | **Insecticide used** | |
| Metselaar [51] | Nothing specific mentioned | | Usually DDT but sometimes dieldrin | |
| Hii [37] | Regular DDT spraying withdrawn 18 months before survey | | DDT | |
| Rowland c)-g) [52] | In Pakistan malathion has been in use, not entirely clear if was sprayed before but assumed it was | | Malathion (f+g), lambdacyhalothrin (c-e) | |
| Sharma [53] | Nothing specific mentioned | | DDT | |
| Rowland i)-p) [31] | Unclear if spraying happened before | | Pyrethroid lambdacyhalothrin (5 villages), malathion (9 villages) | |
| Rowland q)+r) [32] | Unclear if spraying happened before but unlikely | | Alphacypermethrin | |

*^1^Only 10% of population received nets, patent infections of people with nets were compared to infections of people with no nets*

*^2^assumed to be repeated distribution because of high net coverage*

*^3^Only children received nets and were tested*

*^4^Only children received treatment and were tested*

**Most areas had some level of case management*

**References**

1. Ome-Kaius M, Kattenberg JH, Zaloumis S, Siba M, Kiniboro B, Jally S *et al.* Differential impact of malaria control interventions on *P. falciparum* and *P. vivax* infections in young Papua New Guinean children. *BMC Med* 2019;17(1):220.
2. Lin E, Kiniboro B, Gray L, Dobbie S, Robinson L, Laumaea A *et al.* Differential patterns of infection and disease with *P. falciparum* and *P. vivax* in young Papua New Guinean children. *PLOS ONE* 2010;5(2):e9047.
3. Betuela I, Rosanas-Urgell A, Kiniboro B, Stanisic DI, Samol L, de Lazzari E *et al*. Relapses contribute significantly to the risk of *Plasmodium vivax* infection and disease in Papua New Guinean children 1-5 years of age. *J Inf Dis* 2012;206(11):1771-80.
4. Hetzel MW, Reimer LJ, Gideon G, Koimbu G, Barnadas C, Makita L *et al*. Changes in malaria burden and transmission in sentinel sites after the roll-out of long-lasting insecticidal nets in Papua New Guinea. *Parasit Vector* 2016;9(1):340.
5. Kessler A, van Eijk AM, Jamir L, Walton C, Carlton JM, Albert S. Malaria in Meghalaya: a systematic literature review and analysis of data from the National Vector-Borne Disease Control Programme. *Malar J* 2018;17(1):411.
6. Chaves LF, Kaneko A, Taleo G, Pascual M, Wilson ML. Malaria transmission pattern resilience to climatic variability is mediated by insecticide-treated nets. *Malar J* 2008;7:100.
7. Kaneko A, Taleo G, Kalkoa M, Yaviong J, Reeve PA, Ganczakowski M *et al.* Malaria epidemiology, glucose 6-phosphate dehydrogenase deficiency and human settlement in the Vanuatu Archipelago. *Acta Trop* 1998;70(3):285-302.
8. Loha E, Deressa W, Gari T, Balkew M, Kenea O, Solomon T *et al*. Long-lasting insecticidal nets and indoor residual spraying may not be sufficient to eliminate malaria in a low malaria incidence area: results from a cluster randomized controlled trial in Ethiopia. *Malar J* 2019;18(1):141.
9. Shah NK, Tyagi P, Sharma SK. The impact of artemisinin combination therapy and long-lasting insecticidal nets on forest malaria incidence in tribal villages of India, 2006-2011. *PLOS ONE* 2013;8(2):e56740.
10. Rowland M, Bouma M, Ducornez D, Durrani N, Rozendaal J, Schapira A *et al.* Pyrethroid-impregnated bed nets for personal protection against malaria for Afghan refugees. *Trans Roy Soc Trop Med Hyg* 1996;90(4):357-61.
11. Rowland M, Hewitt S, Durrani N, Saleh P, Bouma M, Sondorp E. Sustainability of pyrethroid-impregnated bednets for malaria control in Afghan communities. *Bull WHO* 1997;75(1):23-9.
12. Sahu SS, Jambulingam P, Vijayakumar T, Subramanian S, Kalyanasundaram M. Impact of alphacypermethrin treated bed nets on malaria in villages of Malkangiri district, Orissa, India. *Acta Trop* 2003;89(1):55-66.
13. Maude RJ, Nguon C, Ly P, Bunkea T, Ngor P, Canavati de la Torre SE *et al.* Spatial and temporal epidemiology of clinical malaria in Cambodia 2004-2013. *Malar J* 2014;13:385.
14. Sena LD, Deressa WA, Ali AA. Analysis of trend of malaria prevalence in south-west Ethiopia: a retrospective comparative study*. Malar J* 2014;13:188.
15. Chaumeau V, Kajeechiwa L, Fustec B, Landier J, Naw Nyo S, Nay Hsel S *et al.* Contribution of asymptomatic Plasmodium infections to the transmission of malaria in Kayin State, Myanmar. *J Inf Dis* 2019;219(9):1499-509.
16. Landier J, Kajeechiwa L, Thwin MM, Parker DM, Chaumeau V, Wiladphaingern J *et al.* Safety and effectiveness of mass drug administration to accelerate elimination of artemisinin-resistant falciparum malaria: A pilot trial in four villages of Eastern Myanmar. *Wellcome Open Res* 2017;2:81.
17. Rodriguez-Rodriguez D, Maraga S, Lorry L, Robinson LJ, Siba PM, Mueller I *et al*. Repeated mosquito net distributions, improved treatment, and trends in malaria cases in sentinel health facilities in Papua New Guinea. *Malaria J* 2019;18(1):364.
18. Mitjà O, Paru R, Selve B, Betuela I, Siba P, De Lazzari E *et al.* Malaria epidemiology in Lihir Island, Papua New Guinea. *Malar J* 2013;12:98.
19. Loha E, Lunde TM, Lindtjørn B. Effect of bednets and indoor residual spraying on spatio-temporal clustering of malaria in a village in south Ethiopia: a longitudinal study. *PLOS ONE* 2012;7(10):e47354.
20. Garfield RM, Vermund SH. Changes in malaria incidence after mass drug administration in Nicaragua. *Lancet* 1983;2(8348):500-3.
21. McGreevy PB, Dietze R, Prata A, Hembree SC. Effects of immigration on the prevalence of malaria in rural areas of the Amazon basin of Brazil. *Memorias do Instituto Oswaldo Cruz* 1989;84(4):485-91.
22. Kondrashin AV, Sanyal MC. Mass drug administration in Andhra Pradesh in areas under Plasmodium falciparum containment programme. *J Communic Dis* 1985;17(4):293-9.
23. Landier J, Parker DM, Thu AM, Lwin KM, Delmas G, Nosten FH. Effect of generalised access to early diagnosis and treatment and targeted mass drug administration on *Plasmodium falciparum* malaria in Eastern Myanmar: an observational study of a regional elimination programme. *Lancet* 2018;391(10133):1916-26.
24. Gabaldon A, Guerrero L. An attempt to eradicate malaria by the weekly administration of pyrimethamine in areas of out-of-doors transmission in Venezuela. *Am J Trop Med Hyg* 1959;8(4):433-9.
25. Singh N, Shukla MM, Mishra AK, Singh MP, Paliwal JC, Dash AP. Malaria control using indoor residual spraying and larvivorous fish: a case study in Betul, central India. *Trop Med Int Health* 2006;11(10):1512-20.
26. Seyoum D, Kifle YG, Rondeau V, Yewhalaw D, Duchateau L, Rosas-Aguirre A *et al*. Identification of different malaria patterns due to *Plasmodium falciparum* and *Plasmodium vivax* in Ethiopian children: a prospective cohort study. *Malar J* 2016;15:208.
27. Doke PP, Sathe RS, Chouhan SP, Bhosale AS. Impact of single round of indoor residual spray with lambda-cyhalotrin 10% WP on *Plasmodium falciparum* infection in Akola district, Maharashtra State. *J Communic Dis* 2000;32(3):190-200.
28. Kattenberg JH, Erhart A, Truong MH, Rovira-Vallbona E, Vu KAD, Nguyen THN *et al.* Characterization of *Plasmodium falciparum* and *Plasmodium vivax* recent exposure in an area of significantly decreased transmission intensity in Central Vietnam. *Malar J* 2018;17(1):180.
29. Charlwood JD, Alecrim WD, Fe N, Mangabeira J, Martins VJ. A field trial with Lambda-cyhalothrin (ICON) for the intradomiciliary control of malaria transmitted by Anopheles darlingi root in Rondonia, Brazil. *Acta Trop* 1995;60(1):3-13.
30. Mishra AK, Bharti PK, Kareemi TI, Chand SK, Tidgam AS, Sharma RK *et al.* Field evaluation of zero vector durable lining to assess its efficacy against malaria vectors and malaria transmission in tribal areas of the Balaghat district of central India. *Trans Roy Soc Trop Med Hyg* 2019;113(10):623-31.
31. Rowland M, Hewitt S, Durrani N, Bano N, Wirtz R. Transmission and control of vivax malaria in Afghan refugee settlements in Pakistan. *Trans Roy Soc Trop Med Hyg* 1997;91(3):252-5.
32. Rowland M, Mahmood P, Iqbal J, Carneiro I, Chavasse D. Indoor residual spraying with alphacypermethrin controls malaria in Pakistan: a community-randomized trial. *Trop Med Int Health* 2000;5(7):472-81.
33. Koepfli C, Ome-Kaius M, Jally S, Malau E, Maripal S, Ginny J *et al.* Sustained malaria control over an 8-year period in Papua New Guinea: The challenge of low-density asymptomatic Plasmodium infections. *J Inf Dis* 2017;216(11):1434-43.
34. Thomson R, Sochea P, Sarath M, MacDonald A, Pratt A, Poyer S *et al*. Rubber plantations and drug resistant malaria: a cross-sectional survey in Cambodia. *Malar J* 2019;18(1):379.
35. Rowland M, Webster J, Saleh P, Chandramohan D, Freeman T, Pearcy B *et al*. Prevention of malaria in Afghanistan through social marketing of insecticide-treated nets: evaluation of coverage and effectiveness by cross-sectional surveys and passive surveillance. *Trop Med Int Health* 2002;7(10):813-22.
36. Smithuis FM, Kyaw MK, Phe UO, van der Broek I, Katterman N, Rogers C *et al.* The effect of insecticide-treated bed nets on the incidence and prevalence of malaria in children in an area of unstable seasonal transmission in western Myanmar. *Malar J* 2013;12:363.
37. Hii JL, Kanai L, Foligela A, Kan SK, Burkot TR, Wirtz RA. Impact of permethrin-impregnated mosquito nets compared with DDT house-spraying against malaria transmission by Anopheles farauti and An.punctulatus in the Solomon Islands. *Med Vet Entomol* 1993;7(4):333-8.
38. Deressa W, Yihdego YY, Kebede Z, Batisso E, Tekalegne A, Dagne GA. Effect of combining mosquito repellent and insecticide treated net on malaria prevalence in Southern Ethiopia: a cluster-randomised trial. *Parasit Vector* 2014;7:132.
39. Hetzel MW, Morris H, Tarongka N, Barnadas C, Pulford J, Makita L *et al*. Prevalence of malaria across Papua New Guinea after initial roll-out of insecticide-treated mosquito nets. *Trop Med Int Health* 2015;20(12):1745-55.
40. Hetzel MW, Pulford J, Ura Y, Jamea-Maiasa S, Tandrapah A, Tarongka N *et al*. Insecticide-treated nets and malaria prevalence, Papua New Guinea, 2008-2014. *Bull WHO* 2017;95(10):695-705b.
41. Hetzel MW, Saweri OP, Kuadima JJ, Smith I, Ura Y, Tandrapah A *et al.* Papua New Guinea malaria indicator survey 2016-2017: malaria prevention, infection and treatment. Papua New Guinea Institue of Medical Research, Goroka, 2018; 2018.
42. Luxemburger C, Perea WA, Delmas G, Pruja C, Pecoul B, Moren A. Permethrin-impregnated bed nets for the prevention of malaria in schoolchildren on the Thai-Burmese border. *Trans Roy Soc Trop Med Hyg* 1994;88(2):155-9.
43. Sluydts V, Durnez L, Heng S, Gryseels C, Canier L, Kim S *et al.* Efficacy of topical mosquito repellent (picaridin) plus long-lasting insecticidal nets versus long-lasting insecticidal nets alone for control of malaria: a cluster randomised controlled trial. *Lancet Inf Dis* 2016;16(10):1169-77.
44. Sluydts V, Heng S, Coosemans M, Van Roey K, Gryseels C, Canier L *et al*. Spatial clustering and risk factors of malaria infections in Ratanakiri Province, Cambodia. *Malar J* 2014;13:387.
45. Kattenberg JH, Gumal DL, Ome-Kaius M, Kiniboro B, Philip M, Jally S *et al*. The epidemiology of *Plasmodium falciparum* and *Plasmodium vivax* in East Sepik Province, Papua New Guinea, pre- and post-implementation of national malaria control efforts. *Malar J* 2020;19(1):198.
46. Hofmann NE, Karl S, Wampfler R, Kiniboro B, Teliki A, Iga J *et al.* The complex relationship of exposure to new Plasmodium infections and incidence of clinical malaria in Papua New Guinea. *eLife* 2017; 6: e23708
47. Robinson LJ, Wampfler R, Betuela I, Karl S, White MT, Li Wai Suen CS *et al*. Strategies for understanding and reducing the *Plasmodium vivax* and *Plasmodium ovale* hypnozoite reservoir in Papua New Guinean children: a randomised placebo-controlled trial and mathematical model. *PLOS Med* 2015;12(10):e1001891.
48. Kligler I, Mer G. Periodic intermittent treatment with chinoplasmine as a measure of malaria control in a hyperendemic area. *Revista di Malariologia* 1931;10(4).
49. van DW. Mass treatment of malaria with chloroquine. Results of a trial in Inanwatan. *Trop Geogr Med* 1961;13:351-6.
50. Lwin KM, Imwong M, Suangkanarat P, Jeeyapant A, Vihokhern B, Wongsaen K *et al*. Elimination of *Plasmodium falciparum* in an area of multi-drug resistance. *Malar J* 2015;14:319.
51. Metselaar D. Seven years' malaria research and residual house spraying in Netherlands New Guinea. *Am J Trop Med Hyg* 1961;10:327-34.
52. Rowland M, Hewitt S, Durrani N. Prevalence of malaria in Afghan refugee villages in Pakistan sprayed with lambdacyhalothrin or malathion. *Trans Roy Soc Trop Med Hyg* 1994; 88(4): 378-9.
53. Sharma SN, Shukla RP, Raghavendra K, Subbarao SK. Impact of DDT spraying on malaria transmission in Bareilly District, Uttar Pradesh, India. *J Vector Borne Dis* 2005;42(2):54-60.
