# Supplementary material for: The relative impact of interventions on sympatric Plasmodium vivax and Plasmodium falciparum malaria: A systematic review
Source: PLoS Negl Trop Dis. 2022 Jun 29;16(6):e0010541. doi: 10.1371/journal.pntd.0010541 (PMC9242512; doi:10.1371/journal.pntd.0010541)
Supplement: S3 Supporting Information — (DOCX) [file pntd.0010541.s003.docx]

**S3 Supporting Information: Results from the regression analyses**

Regression analyses of the factors affecting the change in *P. vivax* cases by time since the intervention was implemented

The models were checked for fit graphically and in all cases, they fitted reasonably well. For example, the logistic regression for first time ITN distribution with a random effect by series of time-point (Fig A).


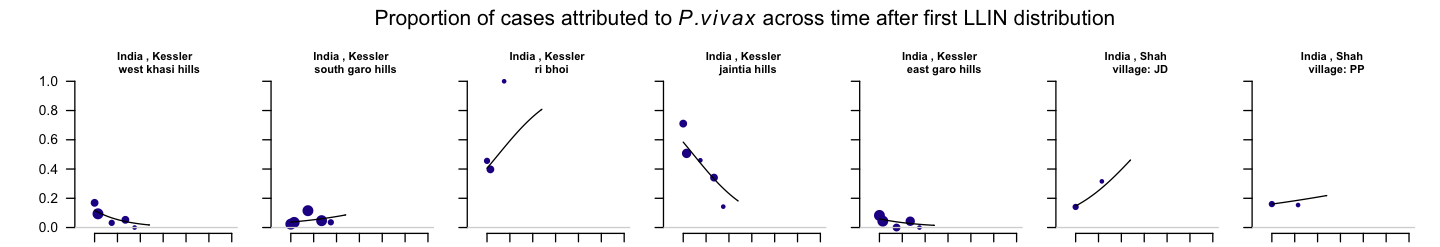


*Figure A. Examples of the fitted proportion over time by the base model (without adjustment for season at time-point of survey) (black lines: fitted lines; circles: observed data)*

**Estimated trend and associations with factors for clinical cases attributed to P. vivax after first time distribution of ITNs**

For the logistic regression model for ITN only data up to 24 months after the intervention was included in the model as the short-term effect of the intervention was of interest.

A logistic regression model was run combining coverage, relapse pattern, transmission intensity as well as initial proportion of *P. vivax*. Seasonality of the setting was not added in the model because the model would not converge. Univariately, seasonality showed neither significance nor a large effect.

*Table A Estimates of the associations between potential factors and the change in the proportion of clinical cases with P. vivax infection by time from first time ITN distribution*

| **Explanatory variable** | **Odds ratio** | **95%CI-lower bound** | **95%CI-upper bound** | **P-value*** |
| --- | --- | --- | --- | --- |
| Base model includes time (adjusted for season of survey°) | | | | |
| Change in odds per month | 1.03 | 1.01 | 1.06 | - |
| Odds ratio for data collected in both seasons | 0.76 | 0.27 | 2.13 |  |
| Odds ratio for data collected in wet seasons | 0.63 | 0.58 | 0.68 |  |
| Model with coverage, relapse pattern, initial proportion of *P. vivax* and transmission intensity  (adjusted for season of survey°) | | | | |
| Change in odds per month in the reference group** | 0.99 | 0.95 | 1.03 |  |
| Extra change per month in high coverage (vs low) | 1.06 | 1.02 | 1.11 | 0.02 |
| Extra change per month missing coverage (vs low) | 1.03 | 0.98 | 1.07 |  |
| Extra change per month with both relapse patterns (vs frequent) | 0.97 | 0.91 | 1.04 | 0.64 |
| Extra change per month long latency relapse patterns(vs frequent) | 0.99 | 0.95 | 1.04 |  |
| Extra change per month with high initial proportion of *Pv* (vs low) | 0.94 | 0.90 | 0.99 | 0.014 |
| Extra change per month with high *Pf* high *Pv* (vs low *Pf* low *Pv*) | 1.02 | 0.97 | 1.07 | 0.001 |
| Extra change per month with high *Pf* low *Pv* (vs low *Pf* low *Pv*) | 1.03 | 0.98 | 1.07 |  |
| Extra change per month with low *Pf* high *Pv* (vs low *Pf* low *Pv*) | 1.15 | 1.08 | 1.23 |  |

*Excludes points 24 months after the intervention. RCTs are included. The effects were estimated using logistic regression, random effects by series of time-points for the intercept and slope of time were included.*

**the p-value stems from a likelihood ratio test, testing for significance of the interaction of time with the respective explanatory variable*

*°Season of survey describes if the incidence was collected during the dry season, wet season or both (reference: dry season). This is not assumed to have a causal effect on the effect of the intervention, however, generally P. vivax incidence is expected to be higher compared to* *P. falciparum during the dry season due to relapses.*

***reference group: low coverage, frequent relapse, low initial proportion of P. vivax, low P. vivax transmission intensity and low P. falciparum transmission intensity.*

In the base model, the odds of a case being attributed to *P. vivax* change by an odds ratio of 1.03 (CI: 1.01-1.06) per month (Table A). This would mean a year after the implementation of the intervention the odds would have increased by a multiple of 1.4 on average. However, there was substantial variation with the estimated increases for the series ranging from an increase of 1.13 to a decrease of 0.92 per month.

In a model containing coverage, transmission, relapse pattern as well as initial proportion of *P. vivax*, there was a significant association between ITN distribution coverage and the change in the proportion of cases that are *P. vivax*. Areas with coverage higher than 70% of the intervention saw a stronger upward trend than those with low coverage.

Compared to areas with low transmission for both *P. vivax* and *P. falciparum* there was a significantly stronger increase with low *P. falciparum* and high *P. vivax* transmission. However, this stems from three series only (Fig 9).

When there is high initial proportion of *P. vivax* there is an estimated downward trend. Relapse pattern was univariately associated with the trend, but was confounded with coverage.

Clinical cases during the wet season tend to have lower odds of being attributed to *P. vivax*. This means in general (also at baseline before the intervention was implemented) there are lower proportions of cases attributed to *P. vivax* during the wet season than during the dry season.


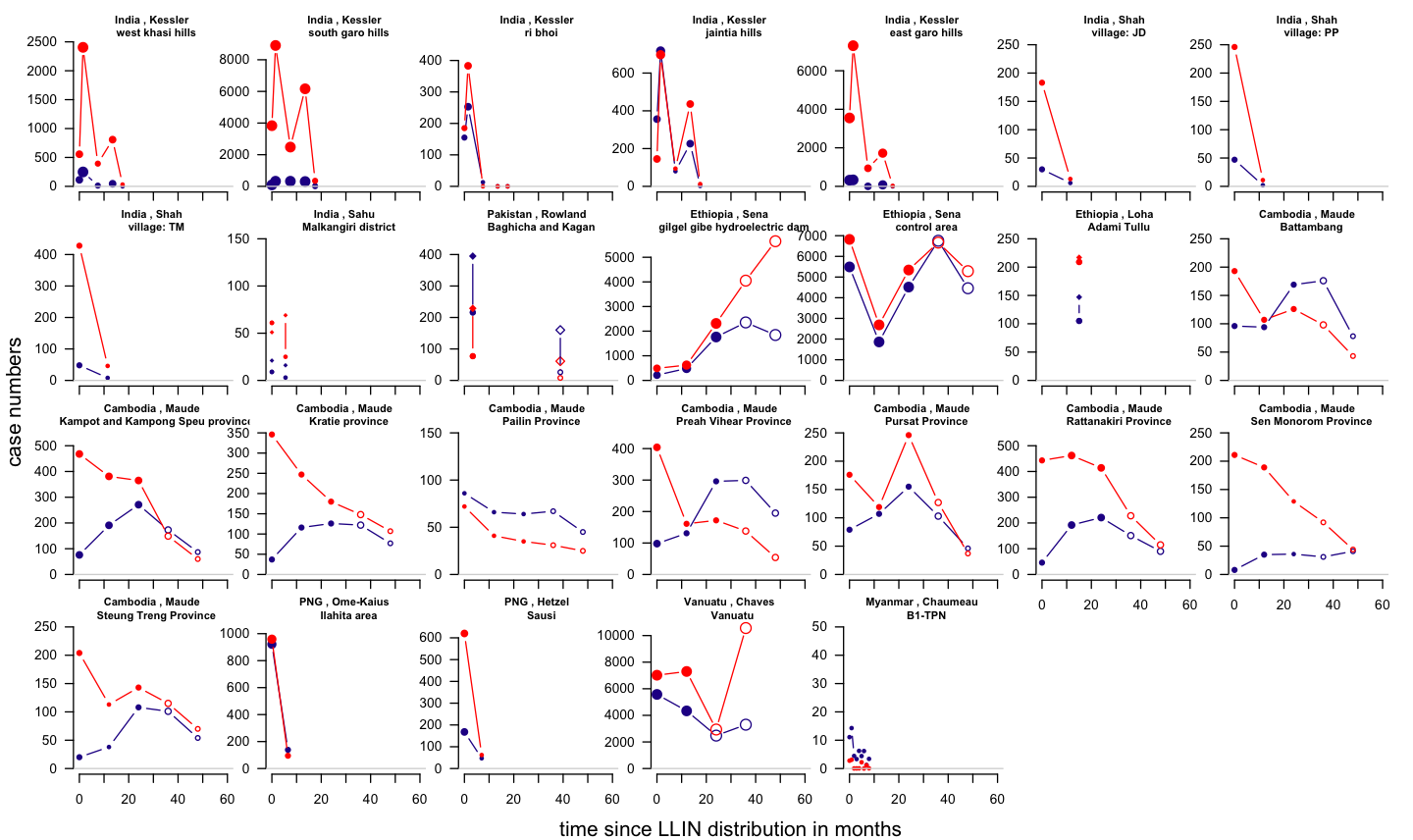


*Figure B. The number of P. vivax cases (blue) and P. falciparum cases (red) over time after first ITN distribution*

The effect of the intervention on the proportion over time could be driven by changes in incidence for both species or one species only (Fig B). However, there was no clear pattern overall In Cambodia *P. falciparum* decreases mostly whereas *P. vivax* increases. Initial increases in Maude [50] might be due to the increased number of village malaria workers (VMWs), if they had a differential impact on detection. In India *P. vivax* cases tend to be low compared to *P. falciparum* and seem to have less fluctuation.

**Estimated trend and associations with factors for clinical cases attributed to *P. vivax* after repeated distribution of ITNs**

Overall, there is an upward trend in the odds of a case being attributed to *P. vivax* over time with a repeated distribution of ITNs (Table B). Long latency relapses lead to a stronger increase, however this is not significant and due to three studies only.

With high *P. falciparum* transmission and low *P. vivax* transmission there is a less pronounced upward trend.

*Table B. Estimates of the association between potential factors and the change in the proportion of clinical cases with P. vivax infection by time from repeated ITN distribution*

| **Explanatory variable** | **Odds ratio** | **95%CI-lower bound** | **95%CI-upper bound** | **P-value*** |
| --- | --- | --- | --- | --- |
| Base model includes time (adjusted for season of survey°) | | | | |
| Change in odds per month | 1.04 | 1.01 | 1.07 | - |
| Odds ratio for data collected in both seasons | 0.25 | 0.06 | 1.01 |  |
| Odds ratio for data collected in wet seasons | 0.94 | 0.84 | 1.05 |  |
| Model with coverage, relapse pattern and transmission intensity (adjusted for season of survey°) | | | | |
| Change in odds per month in the reference group** | 1.06 | 1.03 | 1.09 |  |
| Extra change per month long latency | 1.07 | 0.99 | 1.16 | 0.10 |
| Extra change per month high coverage | 0.99 | 0.95 | 1.02 | 0.13 |
| Extra change per month missing coverage | 0.91 | 0.83 | 1.00 |  |
| Extra change per month high *Pf* low *Pv* | 0.96 | 0.92 | 0.99 | 0.03 |

*Excludes points 24 months after the intervention. The effects were estimated using logistic regression, random effects by series of time-points for the intercept and slope of time were included.*

**the p-value stems from a likelihood ratio test, testing for significance of the interaction of time with the respective explanatory variable*

*°Season of survey describes if the incidence was collected during the dry season, wet season or both (reference: dry season).*

***reference group: low coverage, frequent relapses, low P. vivax transmission and low P. falciparum transmission*

**Estimated trend and associations with factors for clinical cases attributed to *P. vivax* after first time distribution of ITNs compared to repeated distribution of ITNs**

There was no evidence of a difference in the change in odds for a repeated distribution compared to a first round overall (Table C).

*Table C Estimates of the association between potential factors and the change in the proportion of clinical cases with P. vivax infection by time from ITN distribution*

| **Explanatory variable** | **Odds ratio** | **95%CI-lower bound** | **95%CI-upper bound** | **P-value*** |
| --- | --- | --- | --- | --- |
| Base model includes time (adjusted for season of survey°) | | | | |
| Change in odds per month | 1.03 | 1.02 | 1.05 | - |
| Odds ratio for data collected in both seasons | 0.60 | 0.25 | 1.44 |  |
| Odds ratio for data collected in wet season | 0.72 | 0.67 | 0.77 |  |
| Model with round of intervention (adjusted for season of survey°) | | | | |
| Change in odds per month first round of ITN | 1.03 | 1.01 | 1.06 | 0.79 |
| Extra change per month repeated round of ITN | 1.01 | 0.97 | 1.05 |  |
| Model with coverage, relapse pattern and transmission intensity (adjusted for season of survey°) | | | | |
| Change in odds per month in reference group** | 1.00 | 0.96 | 1.04 |  |
| Extra change per month with repeated distribution | 1.05 | 1.00 | 1.11 |  |
| Extra change per month with high coverage | 1.05 | 1.01 | 1.09 | 0.01 |
| Extra change per month with missing coverage | 1.04 | 1.00 | 1.09 |  |
| Extra change per month repeated distribution and high coverage | 0.94 | 0.89 | 1.00 |  |
| Extra change per month repeated distribution and missing coverage | 0.87 | 0.78 | 0.97 |  |
| Extra change per month with long latency and frequent relapses | 0.97 | 0.90 | 1.04 | 0.001 |
| Extra change per month with long latency relapses | 0.96 | 0.92 | 1.01 |  |
| Extra change per month repeated round of ITN and long latency relapse pattern | 1.12 | 1.02 | 1.23 |  |
| Extra change per months with high *Pf* high *Pv* | 0.99 | 0.94 | 1.03 | 0.12 |
| Extra change per months with high *Pf* low *Pv* | 1.03 | 0.98 | 1.08 |  |
| Extra change per months with low *Pf* high *Pv* | 1.12 | 1.05 | 1.19 |  |
| Extra change per month repeated distribution and high *Pf* low *Pv* | 0.94 | 0.88 | 1.00 |  |

*Excludes points 24 months after the intervention. RCTs are included. The effects were estimated using logistic regression, random effects by series of time-points for the intercept and slope of time were included.*

**the p-value stems from a likelihood ratio test, testing for significance of the interaction between the intervention with the respective explanatory variable and time.*

*°Season of survey describes if the incidence was collected during the dry season, wet season or both (reference: dry season). **reference group: first time ITN distribution, low coverage, frequent relapses, low P. vivax transmission and low P. falciparum transmission*

There is a reduced effect estimated for high coverage when there is a repeated round of ITN distribution compared to the first round. This is plausible because with a higher coverage there is a higher impact expected for the first round, but for repeated rounds the coverage may already be high before the distribution. The difference in coverage before and after the distribution may be more important than coverage itself.

There was a significantly different association of relapse pattern by first or repeated round. The increase in the proportion of cases that are *P. vivax* was greater for frequent relapses compared to the long latency pattern for the first round; for the repeated round the long latency relapse pattern had the greater increase.

Seasonality as well as initial proportion of *P. vivax* were not included as in the repeated distributions were all in areas with low seasonality and most showed low initial proportion of *P. vivax* .

**Estimated trend and associations with factors for patent infections attributed to *P. vivax* after distribution of ITNs**

When repeated and first-time distribution patent infection data is put in the same model there are similar results as with clinical cases. The estimated effects are in the same directions for coverage and largely for relapse pattern although for patent infections there is a stronger downward trend in areas with frequent relapses and repeated distribution. Transmission shows partially similar trends. For patent infections there is a tendency for stronger upward trends when *P. vivax* transmission is low for a first time distribution.

Low initial proportion of *P. vivax* leads to stronger upward trends. The effects of initial proportion of *P. vivax* could be confounded with round of intervention as the data from the repeated rounds tend to have lower initial proportions.

*Table D. Estimates of the associations between potential factors and the change in the proportion of patent infections caused by P. vivax by time from ITN distribution*

| **Explanatory variable** | **Odds ratio** | **95%CI-lower bound** | **95%CI-upper bound** | **P-value*** |
| --- | --- | --- | --- | --- |
| Base model includes time (adjusted for season of survey°) | | | | |
| Change in odds per month | 1.02 | 0.98 | 1.06 | - |
| Odds ratio for data collected in both seasons | 1.99 | 1.40 | 2.84 |  |
| Odds ratio for data collected in wet season | 0.66 | 0.41 | 1.05 |  |
| Odds ratio for data collected with missing season | 0.53 | 0.33 | 0.88 |  |
| Model with round of intervention (adjusted for season of survey°) | | | | |
| Change in odds per month first round of ITN | 1.04 | 0.98 | 1.10 | 0.59 |
| Extra change per month repeated round of ITN | 0.98 | 0.90 | 1.06 |  |
| Model with coverage (adjusted for season of survey°) | | | | |
| Change in odds per month first round of ITN low coverage | 0.97 | 0.89 | 1.05 | 0.41 |
| Extra change per month repeated round of ITN | 1.10 | 0.93 | 1.29 |  |
| Extra change per month with high coverage | 1.12 | 1.01 | 1.25 |  |
| Extra change per month with missing coverage | 1.06 | 0.91 | 1.24 |  |
| Extra change per month with high coverage and repeated round of ITN | 0.85 | 0.71 | 1.02 |  |
| Model with relapse pattern (adjusted for season of survey°) | | | | |
| Change in odds per month first round of ITN frequent relapses | 1.10 | 1.06 | 1.14 | <0.001 |
| Extra change per month repeated round of ITN | 0.90 | 0.86 | 0.94 |  |
| Extra change per month long latency relapse pattern | 0.77 | 0.71 | 0.82 |  |
| Extra change per month with long latency relapse pattern and repeated round of ITN | 1.42 | 0.82 | 2.46 |  |
| Model with seasonality (adjusted for season of survey°) | | | | |
| Change in odds per month first round of ITN low seasonality | 1.00 | 0.95 | 1.05 | 0.009 |
| Extra change per month repeated round of ITN | 1.02 | 0.96 | 1.09 |  |
| Extra change per month with high seasonality | 1.14 | 1.04 | 1.25 |  |
| Extra change per month with high seasonality and repeated round of ITN | 0.86 | 0.74 | 0.99 |  |
| Model with transmission intensity (adjusted for season of survey°) | | | | |
| Change in odds per month first round of ITN low *Pf* low *Pv* | 1.16 | 1.01 | 1.34 | 0.0004 |
| Extra change per month repeated round of ITN | 0.85 | 0.74 | 0.99 |  |
| Extra change per month high *Pf* high *Pv* | 0.92 | 0.80 | 1.07 |  |
| Extra change per month high *Pf* low *Pv* | 1.24 | 0.90 | 1.69 |  |
| Extra change per month low *Pf* high *Pv* | 0.75 | 0.65 | 0.87 |  |
| Extra change per month repeated round of ITN and high *Pf* low *Pv* | 0.82 | 0.59 | 1.13 |  |
| Model with initial proportion of *P. vivax* (adjusted for season of survey°) | | | | |
| Change in odds per month first round of ITN and low initial proportion | 1.09 | 0.99 | 1.20 | 0.12 |
| Extra change per month repeated round of ITN | 0.93 | 0.82 | 1.04 |  |
| Extra change per month with high initial proportion | 0.92 | 0.81 | 1.05 |  |
| Extra change per month with repeated round of ITN and high initial proportion | 1.11 | 0.87 | 1.41 |  |

*Excludes points 24 months after the intervention. RCTs are included. The effects were estimated using logistic regression, random effects by series of time-points for the intercept and slope of time were included.*

**the p-value stems from a likelihood ratio test, testing for significance of the interaction of the respective explanatory variable and round of intervention as well as time. For the model with round of intervention only the LRT tests for significance of the interaction of time and round of intervention*

*°Season of survey describes if the incidence was collected during the dry season, wet season or both (reference: dry season).*

**Estimated trend and associations with factors for clinical cases attributed to *P. vivax* after MDA**

In contrast to ITNs the effects of MDA are expected to act on a shorter time scale. Therefore, only time-points within 3 months were used. In this time span the effect of time was assumed to be linear, after checking graphically.

There is a borderline overall downward tendency for the odds of a clinical case being attributed to *P. vivax* in the first three months after the MDA (Table E).

*Table E. Estimates of the association between potential factors and the change in the proportion of cases with P. vivax infection by time from mass drug administration, 0-3 months after MDA*

| **Explanatory variable** | **Odds ratio** | **95%CI-lower bound** | **95%CI-upper bound** | **P-value*** |
| --- | --- | --- | --- | --- |
| Base model includes time (adjusted for season of survey°) | | | | |
| Change in odds per month | 0.93 | 0.63 | 1.39 | - |
| Odds ratio for data collected in wet season | 0.51 | 0.47 | 0.55 |  |
| Model with rounds of MDA (adjusted for season of survey°) | | | | |
| Change in odds per month with one round of MDA | 0.78 | 0.51 | 1.20 | 0.07 |
| Extra change in odds per month with three rounds of MDA | 2.35 | 0.91 | 6.06 |  |
| Model with relapse pattern (adjusted for season of survey°) | | | | |
| Change in odds per month in frequent relapse areas | 3.14 | 1.10 | 8.99 | 0.07 |
| Extra change in odds per month in areas with both relapse pattern | 0.25 | 0.08 | 0.81 |  |
| Extra change in odds per month in areas with long latency relapse pattern | 0.29 | 0.07 | 1.27 |  |
| Model with transmission intensity (adjusted for season of survey°) | | | | |
| Change in odds per month with low *Pf* and low *Pv* transmission | 0.79 | 0.53 | 1.17 | 0.13 |
| Extra change in odds per month with high *Pf* and high *Pv* transmission | 1.70 | 0.58 | 4.99 |  |
| Extra change in odds per month with low *Pf* and high *Pv* transmission | 3.43 | 1.05 | 11.21 |  |
| Model with coverage (adjusted for season of survey°) | | | | |
| Change in odds per month with low coverage | 0.83 | 0.57 | 1.23 | 0.18 |
| Extra change in odds per month with high coverage | 3.30 | 1.00 | 10.83 |  |
| Extra change in odds per month with missing coverage | 1.10 | 0.34 | 3.52 |  |
| Model with initial proportion of *P. vivax* (adjusted for season of survey°) | | | | |
| Change in odds per month with low initial proportion | 0.98 | 0.56 | 1.71 | 0.90 |
| Extra change in odds per month with high initial proportion | 1.05 | 0.49 | 2.28 |  |

*The effects were estimated using logistic regression, random effects by series of time-points for the intercept and slope of time were included.*

**the p-value stems from a likelihood ratio test, testing for significance of the interaction of time with the respective explanatory variable*

*°Season of survey describes if the incidence was collected during the dry season, wet season or both (reference: dry season).*

When three rounds of MDA are done there is an upward trend in the odds of a case being attributed to *P. vivax*. This is also the case for high coverage as well as in areas with frequent relapses and when there is low *P. falciparum* and high *P. vivax* transmission and high *P. falciparum* and high *P. vivax* transmission. As it is mostly the same studies in these categories it is not clear which of these factors would be truly responsible for this upward trend.

When a logistic regression is conducted with the data in the second three months after the intervention the odds of a case being attributed to *P. vivax* increase overall (Table F).

With frequent relapses there is still an estimated upward trend, however for three rounds of MDA, high coverage, high *P. falciparum* and high *P. vivax* transmission as well as low *P. falciparum* and high *P. vivax* transmission there is a downward trend in this second time period. This indicates that the effect of MDA is not linear within six months after the intervention.

*Table F. Estimates of the associations between potential factors and the change in the proportion of cases with P. vivax infection by time from MDA, 3-6 months after the MDA*

| **Explanatory variable** | **Odds ratio** | **95%CI-lower bound** | **95%CI-upper bound** | **P-value*** |
| --- | --- | --- | --- | --- |
| Base model includes time (adjusted for season of survey°) | | | | |
| Change in odds per month | 1.62 | 1.14 | 2.30 |  |
| Odds ratio for data collected in wet season | 0.79 | 0.43 | 1.46 |  |
| Model with relapse pattern and rounds of MDA (adjusted for season of survey°) | | | | |
| Change in odds per month frequent relapses and once round of MDA | 2.83 | 1.17 | 6.82 |  |
| Extra change both relapse patterns | 0.77 | 0.37 | 1.58 | 0.60 |
| Extra change long latency relapse patterns | 0.59 | 0.20 | 1.73 |  |
| Extra change with three rounds of MDA | 0.49 | 0.25 | 0.96 | 0.12 |
| Model with transmission intensity (adjusted for season of survey°) | | | | |
| Change in odds per month with low *Pf* low *Pv* | 1.86 | 1.45 | 2.38 | 0.05 |
| Extra change per month with high *Pf* and high *Pv* | 0.56 | 0.37 | 0.84 |  |
| Extra change per month with low *Pf* and high *Pv* | 0.66 | 0.46 | 0.93 |  |
| Model with coverage (adjusted for season of survey°) | | | | |
| Change in odds per month with low coverage | 1.84 | 1.46 | 2.33 | 0.015 |
| Extra change per month with high coverage | 0.66 | 0.47 | 0.93 |  |
| Extra change per month with missing coverage | 0.58 | 0.45 | 0.75 |  |

*The effects were estimated using logistic regression, random effects by series of time-points for the intercept and slope of time were included. *the p-value stems from a likelihood ratio test, testing for significance of the interaction of time with the respective explanatory variable* °*Season of survey describes if the incidence was collected during the dry season, wet season or both (reference: dry season).*

**Estimated trend and associations with factors for patent infections attributed to *P. vivax* after MDA**

As for the clinical cases, the time period included in the analysis was three months after MDA. Round of MDA was not considered as there was only one study that only had one round.

*Table G. Estimates of the association between potential factors and the change in the proportion of patent infections caused by P. vivax by time from mass drug administration, 0-3 months after the distribution*

| **Explanatory variable** | **Odds ratio** | **95%CI-lower bound** | **95%CI-upper bound** | **P-value*** |
| --- | --- | --- | --- | --- |
| Base model includes time (adjusted for season of survey°) | | | | |
| Change in odds per month | 0.97 | 0.57 | 1.65 | - |
| Odds ratio for data collected in wet season | 2.23 | 1.37 | 3.64 |  |
| Model with relapse pattern (adjusted for season of survey°) | | | | |
| Change in odds per month frequent relapses | 1.07 | 0.54 | 2.12 | 0.13 |
| Extra change in odds per month both relapses patterns | 0.32 | 0.10 | 0.99 |  |
| Extra change in odds per month long latency | 1.25 | 0.52 | 3.01 |  |
| Model with seasonality (adjusted for season of survey°) | | | | |
| Change in odds per month low seasonality | 0.52 | 0.27 | 1.03 | 0.009 |
| Extra change in odds per month high seasonality | 4.10 | 1.47 | 11.44 |  |
| Model with coverage (adjusted for season of survey°) | | | | |
| Change in odds per month with low coverage | 1.77 | 0.91 | 3.45 | 0.04 |
| Extra change in odds per month with high coverage | 0.42 | 0.20 | 0.89 |  |
| Extra change in odds per month with missing coverage | 0.19 | 0.07 | 0.58 |  |
| Model with initial proportion of *P. vivax* (adjusted for season of survey°) | | | | |
| Change in odds per month with low initial proportion | 0.88 | 0.44 | 1.76 | 0.71 |
| Extra change in odds with high initial proportion | 1.19 | 0.45 | 3.20 |  |

*The effects were estimated using logistic regression, random effects by series of time-points for the intercept and slope of time were included.*

**the p-value stems from a likelihood ratio test, testing for significance of the interaction of time with the respective explanatory variable*

*°Season of survey describes if the incidence was collected during the dry season, wet season or both (reference: dry season).*

Similarly to the clinical cases , there is an overall downward trend in the first three months (Table G). The effects of the explanatory variables differ from those seen in the analysis of clinical cases. With high coverage there appear to be downward trends whereas long latency relapse pattern increase the odds of a case being attributed to *P. vivax*.

High seasonality is associated with a strong upward trend. However, this could be due to the data available, for areas with both relapse patterns there is only one series of time-points, in areas with long latency patterns all the series are from one study in Palestine. Furthermore, these three series also have low coverage and high seasonality, both of which are estimated to have strong upward trends over time.

No analysis was done for the time after three months as only five studies would have several time-points after that.

**Estimated trend and associations with factors for clinical cases attributed to *P. vivax* after IRS**

The base model does not predict an increase or decrease in the odds of cases attributed to *P. vivax* over time overall. However, as seen in the spaghetti plot there is substantial variability in the individual series. Frequent relapse pattern was associated with an increase over time, however there was only one study located in a frequent relapse pattern area.

Both IRS and ITN are vector control interventions. Similar to the first-time distribution of nets long latency relapse pattern show less of an increase than frequent relapses.

*Table H. Estimates of the association between potential factors and the change in the proportion of cases with P. vivax infection by time from indoor residual spraying*

| **Explanatory variable** | **Odds ratio** | **95%CI-lower bound** | **95%CI-upper bound** | **P-value*** |
| --- | --- | --- | --- | --- |
| Base model includes time (adjusted for season of survey°) | | | | |
| Change in odds per month | 1.00 | 0.95 | 1.06 | - |
| Odds ratio for data collected in both seasons | 1.29 | 1.19 | 1.40 |  |
| Odds ratio for data collected in wet season | 1.28 | 1.19 | 1.38 |  |
| Model with relapse pattern, seasonality and initial proportion of *P. vivax* (adjusted for season of survey°) | | | | |
| Change in odds per month frequent relapses, low seasonality and low initial proportion of *Pv* | 1.41 | 1.13 | 1.75 |  |
| Extra change per month low and frequent | 0.71 | 0.58 | 0.87 | 0.015 |
| Extra change per month long latency | 0.70 | 0.56 | 0.87 |  |
| Extra change per month high seasonality | 1.02 | 0.92 | 1.13 | 0.69 |
| Extra change per month high initial proportion of *Pv* | 0.96 | 0.88 | 1.04 | 0.38 |

*Only data up to 24 months after the intervention. The effects were estimated using logistic regression, random effects by series of time-points for the intercept and slope of time were included. RCTs included.*

**the p-value stems from a likelihood ratio test, testing for significance of the interaction of time with the respective explanatory variable °Season of survey describes if the incidence was collected during the dry season, wet season or both (reference: dry season).*

**Estimated trend and associations with factors for patent infections attributed to *P. vivax* after IRS**

For patent infections following indoor residual spraying, there is an overall upward trend in the odds of a case being attributed to *P. vivax* (Table I).

In a model combining transmission, seasonality and initial proportion of *P. vivax*, transmission shows strong point estimates for the effect over time although these are not significant. There were only two studies with high transmission for both species and hence this estimate could be due to the studies available.

*Table I. Estimates of the association between potential factors and the change in the proportion of patent infections caused by P. vivax by time from indoor residual spraying*

| **Explanatory variable** | **Odds ratio** | **95%CI-lower bound** | **95%CI-upper bound** | **P-value*** |
| --- | --- | --- | --- | --- |
| Base model includes time (adjusted for season of survey°) | | | | |
| Change in odds per month | 1.27 | 1.08 | 1.51 | - |
| Odds ratio for data collected in wet season | 0.60 | 0.32 | 1.14 |  |
| Odds ratio for data collected with missing seasons | 0.45 | 0.15 | 1.30 |  |
| Model with transmission intensity, seasonality and initial proportion *P. vivax*  (adjusted for season of survey°) | | | | |
| Change in odds per month low *Pf,* low *Pv*, low seasonality and low initial proportion of *Pv* | 1.30 | 0.94 | 1.78 |  |
| Extra change per month high *Pf* and high *Pv* | 0.78 | 0.56 | 1.07 | 0.13 |
| Extra change per month low *Pf* and high *Pv* | 1.36 | 0.94 | 1.96 |  |
| Extra change per month high seasonality | 0.92 | 0.68 | 1.25 | 0.59 |
| Extra change per month high initial proportion of *Pv* | 0.86 | 0.59 | 1.26 | 0.43 |

*Only data up to 24 months after the intervention. The effects were estimated using logistic regression, random effects by series of time-points for the intercept and slope of time were included. RCTs included.*

**the p-value stems from a likelihood ratio test, testing for significance of the interaction of time with the respective explanatory variable*

*°Season of survey describes if the incidence was collected during the dry season, wet season or both (reference: dry season).*

**Estimated trend and associations including all interventions on one model**

In a model containing all interventions together, there was no significant evidence of a different trend over time by intervention type.(Table J). In the case of patent infections (Table K), this was borderline significant with a reduced increase for repeated compared to a first-time net distribution.

The effects of the covariates appeared to differ by intervention, but could not be fitted together due to convergence issues.

*Table J. Estimates of the association between potential factors and the change in the proportion of cases with P. vivax infection by time from the intervention implementation*

| **Explanatory variable** | **Odds ratio** | **95%CI-lower bound** | **95%CI-upper bound** | **P-value*** |
| --- | --- | --- | --- | --- |
| Model including time (adjusted for season of survey°) | | | | |
| Change in odds per month in with first time ITN distribution | 1.04 | 0.97 | 1.11 | 0.34 |
| Extra change per month with repeated ITN distribution | 0.98 | 0.87 | 1.10 |  |
| Extra change per month with IRS | 0.96 | 0.87 | 1.07 |  |
| Extra change per month with MDA | 0.87 | 0.75 | 1.01 |  |
| Odds ratio for data collected in both seasons | 0.79 | 0.75 | 0.83 |  |
| Odds ratio for data collected in wet season | 0.79 | 0.76 | 0.82 |  |

*Data included up to 24 months after the intervention for IRS and ITNs, for MDA up to 3 months after the intervention. RCTs included*

*°adjusted for season at time-point of survey*

**the p-value stems from a likelihood ratio test, testing for significance of the interaction of time with the type of intervention*

*Table K. Estimates of the association between potential factors and the change in the proportion of patent infections caused by P. vivax by time from the intervention implementation*

| **Explanatory variable** | **Odds ratio** | **95%CI-lower bound** | **95%CI-upper bound** | **P-value*** |
| --- | --- | --- | --- | --- |
| Model including time (adjusted for season of survey°) | | | | |
| Change in odds per month in with first time ITN distribution | 1.06 | 0.96 | 1.16 | 0.07 |
| Extra change per month with repeated ITN distribution | 0.95 | 0.83 | 1.09 |  |
| Extra change per month with IRS | 1.14 | 0.97 | 1.35 |  |
| Extra change per month with MDA | 0.83 | 0.64 | 1.07 |  |
| Odds ratio for data collected in both seasons | 1.80 | 1.25 | 2.60 |  |
| Odds ratio for data collected season in wet season | 1.42 | 1.00 | 2.00 |  |
| Odds ratio for data collected with missing season | 1.21 | 0.61 | 2.40 |  |

*Data included up to 24 months after the intervention for IRS and ITNs, for MDA up to 3 months after the intervention. RCTs included*

*°adjusted for season at time-point of survey*

**the p-value stems from a likelihood ratio test, testing for significance of the interaction of time with the type of intervention*
