# Supplementary material for: The relative impact of interventions on sympatric Plasmodium vivax and Plasmodium falciparum malaria: A systematic review
Source: PLoS Negl Trop Dis. 2022 Jun 29;16(6):e0010541. doi: 10.1371/journal.pntd.0010541 (PMC9242512; doi:10.1371/journal.pntd.0010541)
Supplement: Attachment — Submitted filename: response_to_reviewers.pdf [file pntd.0010541.s004.pdf]

Response to reviewers' comments

We thank the reviewers for their helpful comments. We have added them anonymously to the Acknowledgements section.

Throughout this document, the line numbers refer to the version with tracked changes.

Methods

Reviewer #1: Objectives and methods clearly described in detail, but statistical analysis may benefit from an expert biostatistician with experience in the field.

One of the authors is a biostatistician with experience in malaria epidemiology. We initially did the analysis in two ways: as an individual participant data analysis as presented here and as a meta-regression combining the estimated odds ratio per month since the implementation of the intervention and standard error from each study. We present the individual participant analyses since this is recommended where the individual data are available or can be extracted. The meta-regression did not provide any additional information and lead to the same conclusions.

The analysis approach was tailored to the specific requirements of this review. The outcome (the proportion of cases that were attributed to P. vivax) is binary and so we used logistic regression. Our focus was the change in the proportion of vivax cases over time since the implementation and so we included a slope (parameterizing the change as the odds ratio per month). The effect of the covariates was allowed to affect both the intercept and slope, but it was the effect on the slope that was of particular interest for this study. We included random effects for series to account for the clustering of the data within separate series. We have now clarified this in the Analysis section.

We analysed the interventions separately, because our hypotheses were that they would lead to different patterns over time from implementation and because the follow-up periods and amount of data differed by intervention. A combined analysis lead to the same conclusions.

Reviewer #2: The objectives of this study were to conduct an analysis of the published literature reporting on the effect of three malaria control interventions implemented in regions co-endemic for Plasmodium falciparum and P. vivax in order to estimate the proportions of clinical cases and patent infections attributable to P. vivax after the implementation.

The methods used were a literature search of publications describing implementation of the three control methods; the proportion of P. vivax infections, either clinical or patent, was calculated by dividing the number of such P. vivax cases by cases of either P. vivax or P. falciparum. The team also looked at the impact on proportion of P. vivax according to different variables. The team is to be commended on such an undertaking, and in the reading on the manuscript, it become clear the challenges encountered with variety of data and methodologies used and in which the primary objective was not to determine proportion of P. vivax infections. The objectives were articulated and study design appropriate. The populations in the manuscript were clearly defined in the Supplemental sections, and appropriate for the hypotheses. The section describing the variables was very clear and well written. There are no ethical or regulatory concerns, and my background does not allow for a critical review of the statistical methods used.

We thank the reviewer.

One question: I saw some studies were from 40 years ago (especially in clinical cases after MDA) and/or prior to widespread implementation to ACTs (CQ used in a few of the patent studies after MDA). I would add the range of year of the studies in the main text, perhaps for each intervention as well. Did the authors consider this factor at all in their own analysis or did they notice any trends that are not reported here?

We have added the range of years of the studies by intervention and outcome (L409 and L426). For most analyses, most series were either pre- or post-2000 and an analysis by time period would be difficult. For MDA clinical cases however, there were three studies that were post-2000 with four series of time-points: the newer studies tended to have a stronger increase but they also tended to have higher coverage, three rounds rather than one round and longer follow-up periods. We have mentioned time period as a potential confounder (L688).

Results

Reviewer #1: The presentation of results is a bit tedious and overwhelming, particularly when describing figures 3 - 20. To avoid distractions and sole description of the graphs, the authors may benefit of rearranging the presentation and transferring these figures to supplemental material. It will require a little creativity to still briefly describe those results in the main text, while highlighting the overall "big picture", which is shown in figure 21. All the other tables and boxes from the main text are important are clearly presented and should be kept as they are.

We recognize that the paper has many figures. However we would like allow the studies themselves (figures 3-18) to share the focus with figure 19 (previously figure 21). Figure 19 is a summary of the regression analyses which quantifies the overall trend and investigates the roles of the covariates. Figures 3-20 summarize the information in turn for each intervention, round and outcome (clinical cases or patent infections) and are the heart of the paper.

We have tried to reduce repetition by editing the results section to reduce the number of sections: we have combined the graphs and text for the descriptions of the first-time and the repeated distributions for each intervention. We have also distilled the information down by further editing of the text (Results, throughout).

Reviewer #2: The results were complete, of good quality clearly presented, and quite informative. The Figures accurately reflected the results and the complexity thereof. In Fig 3-9 graphs, I liked the use of differently sized circles in addition to proportion of cases on Y-axis. I was curious about effects on sheer numbers.

We thank the reviewer.

A few minor points/edits

Figure 3. In the legend it says "Dots at time-point 0 were not necessarily measured at this time but at any time-point before the intervention…Only one pre-implementation point was available", but in some of the graphs there are multiple points at Time zero (graph a for example). Is it the scale of the X-axis? It also states Blue filled circles: proportion of cases with P. vivax infection". Are these numbers raw numbers of P. vivax cases or P. vivax cases divided by Pv/Pf?

We have removed the statement that only one pre-implementation point was available, and also added a reference to the Methods section where the pre-implementation points are explained (Figure legends, Figure 3). We have also checked the section in the Methods where this is explained for clarity (L254), and added a reference to the Methods for figure legends 4-7.

We initially plotted the graphs with the actual time of the pre-implementation time-points, but this potentially gave a misleading impression of a gradual slow change rather than a change after the intervention, and was difficult to compare between series. The median time point of data collection of the pre-implementation time points was 0.2 months before the intervention in case of clinical cases, and 9.7 months for patent infections. All regression analyses were adjusted for the season of measurement.

The definition of the proportions for the outcome variables are given in the Methods (definition of variables, L320).

Figure 4 and 7-9. There are multiple data points at Time zero. Are there different populations within the study i.e., different villages? Or what do they generally represent?

In the graphs of individual series of time-points (figures 3-7), only one village or area is represented in each panel. Studies contributing multiple series have multiple panels. We have added to the legend of each graph a reference to the place in the Methods where the pre-implementation time-points are explained.

Conclusions

Reviewer #1: The conclusions should more clearly state that such high variability in outcomes did not identify a significant factor influencing prevalence of vivax post-intervention. "Trends" can be overseen as indicator of prediction, so words should be carefully chosen in the abstract and conclusions. Limitations were clearly described and the study will be useful for epidemiologists in the field and governing agencies responsible control measures.

We have altered the wording where there is potential for confusion by either using a different word for trends (changes/patterns), or referring to 'observed trends'. We have also clarified that the high variability and limited data available did not allow factors to be ruled in or out (L862).

Reviewer #2: The conclusions reached were that in the short term, 2 years for ITN and IRS, and MDA for >/+ 3 months, an increase in proportion of cases due to P. vivax is seen, albeit with extensive variability among studies.

The data and analysis are helpful in highlighting the complexities of these type of studies on how the variables can effect each intervention differently, in particular the relapsing patterns, which may have more implications for a country such as India or Brazil, which may not have the homogenous fast relapsing patterns of Southeast Asia.

The authors make a nice comparison that this is an analysis different from the long-term analysis of Price et al. The long-term effects of control on proportionality are quite evident in GMS where >75%

of cases are now P. vivax in each country (except Vietnam, although there were less than 1,500 case total in 2021). The authors do state "…change in species composition should be monitored". This point is crucial, and according to the heat map, as are patterns and transmission intensity. The authors go on to advocate for "including more studies in the analysis as they become available… and to describe interventions in detail.." This paper looks back at what has been done, but from here on out, household ITNs and IRS may no longer be the best modalities for all co-endemic countries, and MDA strategies will need to change.

Can the authors expound on what study designs could look like, what objectives those studies should embody, and what interventions can best be used or adapted in the near future for control a shifting epidemiological situation.

We agree that ITN and IRS may no longer be the best modalities for all co-endemic countries, although we think what the best modalities would be is outside the scope of this paper. We have revised this sentence to say that including more studies would be helpful but we recognize that these interventions may not be the most appropriate for all co-endemic settings as the transmission levels decline (L849).

Editorial and Data Presentation Modifications

Reviewer #1: See comments about Results and general comments to the authors.

Reviewer #2: In Figure 4 in the legend there is a misspelled word: Open circles are data points more than 24 months after the intervention and where excluded for the analysis.

This sentence has been edited (now Figure 3).

Meaning of sentence in Line 780 was not clear. There was no evidence of a change in clinical case attributed to P. vivax over time was estimated for IRS.

This sentence has now been deleted (now L784).

Summary and General Comments

Reviewer #1: The manuscript by Melanie Loeffel & Amanda Ross describes a thorough review of the available literature on the impact of three major interventions (treated bednets, ITN; indoor residual spraying, IRS; mass drug administration, MDA) in areas of sympatric vivax and falciparum malaria. The ability to compare dozens of studies side by side as the authors present is of great value to scientists in the field and policy makers when considering future control initiatives.

The approach and methods are clearly described in detail, but the presentation of results is a bit tedious and overwhelming, particularly when describing figures 3 - 20. To avoid distractions and sole description of the graphs, the authors may benefit of rearranging the presentation and transferring these figures to supplemental material. It will require a little creativity to still briefly describe those results in the main text, while highlighting the overall "big picture", which is shown in figure 21. All

the other tables and boxes from the main text are important are clearly presented and should be kept as they are. The interesting finding of the analysis is the great variability observed on vivax malaria prevalence post-intervention. However, such variability highlights caution against over interpretation of the data from figure 21.

This comment was repeated: we have addressed it above (under 'Results').

Finally, the discussion section can be improved to reduce repetition of statements used in the results and to highlight the importance to take into consideration the biological differences between vivax and falciparum when evaluating a general intervention not targeting hypnozoites.

We have edited the Discussion to avoid repetition (from L771). We have added the sentence on the importance of considering the biological differences between the species (L834).

Below are some specific points for consideration:

1. Page 4, line 104: please explain the discrepancy between "973 hits" and "1157" hits described in page 14, line 361.

We have now explained that 973 hits came from PubMed and 184 from screening citations in the Cochrane reviews and the included papers, yielding 1157 hits in total (L378).

2. Page 5, line 112: did the authors find lots of reports in Chinese or Portuguese that had to be discarded due to language? These are regions where Pv and Pf occur and many studies are published only in magazines available to their countries and their languages.

The literature search found 106 studies that were excluded because they were in languages other than English or German. Forty-four were in Chinese and 15 in Portuguese (the others were in Turkish, Hebrew, Russian, Italian, French, Spanish, Polish, Croatian, Hungarian, and Japanese). It is not known if these studies would have been possible to include (they might not have measured the malaria species separately, have included more than one time point, or have provided sufficient information about the intervention.

3. Page 11, lines 281-285: please spell out or quote the definition of relapse patterns described in reference 27. The cited review article has an enormous amount of information and analysis of relapses, so it will be helpful to read the definition the authors decided to use. For example, figure 22 of reference 27 highlights the possibility of confusion caused by similar relapse patterns with long-latency and frequent relapsing parasites.

We have clarified this (now L292). The broad geographical regions in figure 17 of White are used: corresponding to frequent, long latency or both long latency and frequent relapse.

4. Page 12, line 303: to help the readers the authors should write the minimum sample size of the studies and refer to which supplemental material shows the extracted data.

The range of the number of cases per time-point is now given in L420 and L436. The sample size for each time-point is represented in the figures (3-18) by the size of the dots. The total number of cases or infections per series is included in supplement 1 tables S1.2-S1.10. (L303 was in a section of the Methods which describes the data to be extracted, but the data itself is described in the Results section).

5. Page 23, Figure 3: the figure font is too small, but if the authors transfer the graphs to supplemental data they can be presented in full size. To facilitate visualization of the different regions, the titles could be shorter to include just country and city/region, and a background color coded by country. That would quickly show the patterns observed in Cambodia (Fig3 n- v), where primaquine is rarely provided due to high prevalence of G6PD deficiency. This could explain the steady prevalence of vivax malaria and is supported by the fact that most vivax cases following chloroquine treatment in Cambodia are associated with hypnozoite reactivation (Popovici J et al, JID 2019).

We have now submitted the figures separately as full page landscape figures, also because PLOS NTD require them separately to the text for resubmissions. We have reduced repetition by combining the first-time and repeated distributions of ITN (and IRS) in the same graphs and used shading to indicate the round. We have ordered the panels by country and study.

6. Page 24, Figure 4: graph titles are too long, therefore cut in some cases. Please check if the axis title is correct "time since LLIN distribution in months"

We have corrected the graph titles (now Figure 3).

7. Page 25, Figure 5: please clarify if the studies with control groups suggest no difference in the impact of intervention on vivax prevalence.

There were only two studies with both control groups and before and after time-points (now Figure 4 o) and k)). The two comparisons would produce approximately the same conclusions for these two studies, but there is very little data. We have added a sentence on this (L541).

8. Page 27, MDA: considering the important biological difference between vivax and falciparum regarding development of dormant liver stage in the former, the authors should emphasize that drug interventions focus mostly against blood stages. This is critical as relapses cases will account for greater vivax prevalence post-intervention.

We have added a sentence to say that the MDA drug regimens were focused on clearing blood-stage parasites only (L562).

9. Minor text edits should be considered in the lines listed below and a text search and replace should be performed to include a space between "P." and "vivax"/"P." and "falciparum":

- Line 55: spell out Plasmodium vivax instead of P. vivax
- Line 247: missing period after P from P vivax.
- Line 289: space after seasonality and before comma

- Line 529: “…were had received…”
- Line 540: “…to rid of…”
- Line 598: “…there it was likely that…”
- Line 765: “signifiicant”

These have all been corrected.

Reviewer #2: (No Response)
